# Comprehensive discovery and functional characterization of diverse prophages in the pig gut microbiome

**DOI:** 10.3389/fmicb.2025.1662087

**Published:** 2025-09-09

**Authors:** Chao Wei, Yaxiang Wang, Zhe Chen

**Affiliations:** National Key Laboratory of Pig Genetic Improvement and Germplasm Innovation, Jiangxi Agricultural University, Nanchang, China

**Keywords:** prophages, prophage-host dynamics, auxiliary metabolic genes, antibiotic resistance genes, virulence factors, pig guts

## Abstract

Prophages, viruses integrated into bacterial or archaeal genomes, can carry cargo that confers beneficial phenotypes to the host. The porcine gut microbiota constitutes a complex, dynamic, and interconnected ecosystem, yet the distribution of prophages and their unique functional characteristics within this microbial community remains poorly understood. In this study, we identified 10,742 prophage genomes through systematic screening of 7,524 prokaryotic genomes from porcine gut sources, representing both bacterial and archaeal lineages, with the distribution of integrated prophages exhibiting pronounced heterogeneity across host species. Additionally, 1.70% (183/10,742) of prophages exhibited a broad host range infectivity, while 5.07% (545/10,742) of integrated prophages enhanced prokaryotic adaptive immune capabilities by augmenting or directly providing host defense mechanisms. Notably, within tripartite phage-phage-host interactions network analysis, we observed that these prophages (*n* = 15) exhibit preferential acquisition of exogenous invasive phage sequences through CRISPR spacer integration mechanisms. Functional annotation revealed that prophage-encoded integrases and tail tube proteins may be critical determinants of phage host specificity. In addition, key auxiliary metabolic genes are encoded in the prophage of the pig intestinal tract, such as those promoting the synthesis of host microbiota-derived vitamin B12, encoded antibiotic resistance genes, and virulence factors that provide the host with a survival advantage. Furthermore, comparative analysis with existing viral and phage sequences uncovered a substantial reservoir of high-quality novel prophage sequences. Our findings systematically investigated the diversity of prophages in the pig gut, further characterizing their host range, functional attributes, and interactions with both host bacteria and other phages, through large-scale analysis of porcine gut microbiota genomes. This work offers new insights into the ecological roles of prophages and provides valuable genomic resources for studying prophages in this ecosystem.

## 1 Introduction

Phages are the most abundant entities (Clokie et al., 2011) in natural environments and play crucial ecological roles due to their vast abundance and immense diversity (Schulz et al., 2020; Dance, 2021). Their predation of bacteria and archaea has a strong influence on microbial populations within diverse ecosystems (Chevallereau et al., 2022). Phages are classified as lytic or lysogenic life cycles. Lytic phages initiate a productive replication cycle upon infecting prokaryotic hosts, culminating in rapid lysis and exerting substantial regulatory influence on host population density dynamics (Jansson and Wu, 2022). In contrast, lysogenic phages are capable of integrating their genetic material into the host genome following infection, establishing a dormant prophage state that persists without causing immediate host cell lysis (Chevallereau et al., 2022). Prophage, phage sequences integrated into bacterial or archaeal genomes, can be beneficial, yet also pose a lethal threat as they can reactivate and enter a lytic cycle (Sutcliffe et al., 2023). Due to the convertible lifestyle characteristic of the prophage state, it often poses challenges to prophage research.

Several studies (Bondy-Denomy and Davidson, 2014; Feiner et al., 2015; Harrison and Brockhurst, 2017; Howard-Varona et al., 2017) have demonstrated the beneficial roles of prophage, particularly under diverse environmental stresses. During the prophage stage, integrated phage can expand the functional gene repertoire available to the host prokaryotic cell through lysogenic conversion, thereby enhancing the host’s adaptive capacity (Yi et al., 2023). Prophages serve pivotal roles in microbial interaction networks, where their integration facilitates horizontal gene transfer among prokaryotes and confers selective advantages to their hosts (Hu et al., 2021). For instance, numerous prophages have been identified in deep-sea environments (Hurwitz and U’Ren, 2016; Warwick-Dugdale et al., 2019), which can modulate gene expression in marine bacterial hosts, facilitating their adaptation to these extreme habitats. Similarly, Liao et al. (2024) discovered that prophages in the human gut microbiome harbor a substantial number of antibiotic resistance genes (ARGs), highlighting their potential role as an underappreciated reservoir of ARGs. Additionally, a substantial number of prophages have been identified in certain pathogenic bacteria (Pei et al., 2024), and these prophages significantly influence the host’s physiology, metabolism, and virulence. Therefore, phage-host dynamics can serve as a proxy for ecological functions in response to their environmental conditions (Piel et al., 2022; Kauffman et al., 2022). Helpfully, prophages can also confer resistance to infection by related phages upon their bacterial hosts, although the breadth of this resistance varies (Bondy-Denomy et al., 2016). While the benefits of prophages are evident, they also impose costs. The expression of viral proteins during lysogenic conversion can place a metabolic burden on the host, rendering the prophage disadvantageous under certain environmental conditions (Wendling et al., 2021). We still lack large-scale genomic data to better understand prophage activity and function, as well as their impacts on host behavior.

Swine, as an ideal biomedical model, holds significant implications for both agricultural production and human health (Rao et al., 2023). The pig gut microbiome constitutes a complex, dynamic, and interconnected ecosystem (Chen et al., 2021a; Chen et al., 2021b; Yang et al., 2022), it is closely associated with various phenotypic traits of pigs (Fu et al., 2021; Chen et al., 2022). Beyond the bacterial component, increasing attention has been given to the pig gut virome. For example, Hu et al. (2024b) investigated the gut phage composition of 112 individuals from seven different pig breeds and characterized the antibiotic resistance genes carried by these phages. Yu et al. (2025) compared the gut virome composition of mice, pigs, and ynomolgus macaques. Shkoporov et al. (2022) examined the extent of virome sharing across different gut regions. More recently, Mi et al. (2024) established the largest current pig gut virome database (PVD), providing an important resource for future studies. Moreover, pigs are generally raised under intensive farming conditions, which, compared with studies on the human gut virome, reduces the dietary variability that can introduce noise into microbial community analyses (Yang et al., 2022). This allows for a more accurate representation of the natural distribution of prophages in the mammalian gut. In addition, studies of the pig gut virome facilitate the investigation of prophage distribution across the entire gastrointestinal tract, rather than being limited to fecal samples (Shkoporov et al., 2022). Consequently, it offers an excellent model for studying the characteristics of prophages in the mammalian gut and their interactions with the host microbiota. It will facilitate an enhanced understanding of the evolutionary characteristics and life strategies of intestinal prophages.

In this study, we aim to delineate a comprehensive prophage landscape within the porcine intestinal tract and conduct an in-depth investigation into the host range properties, functional characteristics, and interactions of prophages with their microbe hosts and other phages. We obtained 10,742 prophage genomes after systematically screening 7,524 prokaryotic genomes derived from porcine gut sources, encompassing both bacterial and archaeal lineages. Subsequently, we determined their potential host range via a CRISPR spacer-targeting approach, revealing the potential for inter-prokaryotic phage transmission. Annotation of defense systems across all prophage genomes revealed that pig gut prophages possess the potential to aid their hosts in countering infections by other phages, particularly by influencing the integrity of the host CRISPR-Cas systems. Simultaneously, through in-depth analysis of all prophage-encoded proteins in the pig gut, we characterized the distribution of auxiliary metabolic genes (AMGs), antibiotic resistance genes (ARGs), and virulence factors (VFs) within the prophage genomes, as well as their potential roles in shaping microbial hosts. Comparison with public databases revealed that the prophage genomes we identified exhibit high novelty, indicating that we provide valuable new prophage sequence resources. Overall, our study reveals the diversity, ecology, evolution, and functional significance of pig gut-derived prophages, contributing to an enhanced understanding of the roles played by prophages within the pig gut.

## 2 Materials and methods

### 2.1 Genome collection of pig gut-derived prokaryotic genomes

We first collected available genomes (clearly identified as the source of pig intestines or feces) from the National Center for Biotechnology Information database (NCBI, February 2025), three other studies about pig gut microorganisms (Holman et al., 2021; Hu et al., 2024a; Yang et al., 2024), and our laboratory collections. Next, CheckM (Parks et al., 2015) (v1.1.3) was used to evaluate the quality of pig gut prokaryotic genomes, and only high-quality genomes (completeness ≥90% and contamination ≤5%) were retained. Furthermore, taxonomic classification of retained genomes was performed by GTDB-Tk (Chaumeil et al., 2022) (v1.3.0) using the “classify_wf” pipeline. Ultimately, our study compiled a comprehensive collection of 7,524 pig gut-derived prokaryotic genomes, comprising 84 genomes from the NCBI, 2,746 from three other published studies about pig gut microorganisms, and 4,694 generated in our laboratory. The prokaryotic genomes dataset consisted of 7,436 bacterial genomes and 88 archaeal genomes, collectively classified into 799 species, 670 genera, 148 families, 67 orders, 27 classes, and 22 phyla. Detailed information is provided in Supplementary Table S1.

### 2.2 Prophage prediction, genome quality assessment, and taxonomy assignment

We first used VirSorter2 (Guo et al., 2021) (v2.2.2) to predict prophage sequences in the curated pig gut prokaryotic genome dataset with the “--include-groups dsDNAphage, ssDNA --min-length 5,000 --min-score 0.5” and “--include-groups RNA --min-length 1,000 --min-score 0.5” parameter. After conducting targeted searches for RNA-dependent RNA polymerase (RdRP) proteins to further identify RNA phages, we did not find any RNA phages (Dominguez-Huerta et al., 2022; Neri et al., 2022). Next, to remove contaminating bacterial and archaeal sequences, the authenticity of all putative prophages was evaluated based on the bacterial or archaeal universal single-copy orthologs [BUSCO (Waterhouse et al., 2018)] and the curated viral protein family modules [VPFs (Paez-Espino et al., 2017)]. Briefly, proteins encoded by each prophage were searched against the 318 BUSCO gene HMMs with hmmsearch (-E 0.05), and then used the BUSCO-provided HMM score cut-offs to filter the results for “hits.” The rate of BUSCO hits per total number of genes in each Viral RefSeq genome (BUSCO ratio) was assessed, and this established a range of BUSCO ratio values of 0–0.067 that were derived from known virus genomes (Gregory et al., 2020). Meanwhile, to assess the level of viral gene enrichment, an HMMsearch of all putative prophage genomes against VPFs was performed, with hits being defined as any matches with an *e*-value <0.05. The prophage genomes that had a BUSCO ratio <0.067 or had a BUSCO ratio >0.067 and at least 3 VFP hits were retained for further analysis. Furthermore, we utilized geNomad (Camargo et al., 2023) (v1.7.4) with default parameters to remove putative plasmid sequences from these putative prophages. The genome quality of prophages was evaluated using the software CheckV (v. 1.0.1) with default parameters and databases. For not-determined prophage sequences, we further used geNomad with default parameters to assess and only retained theseprophage sequences classified as “Virus.” Finally, 10,742 prophages were obtained, and prophage taxonomy was predicted using geNomad with default parameters.

### 2.3 Acquisition of the CRISPR spacers and alignment with prophages

We utilized MinCED (v0.4.2, https://github.com/ctSkennerton/minced) to predict CRISPR systems among all 7,524 prokaryotic genomes in this study, identifying a total of 44,425 spacer sequences, including 44,063 spacers derived from prokaryotic hosts and 362 spacers from prophages. Then, the BLASTn (v.2.12.0) (Altschu et al., 1990) alignments were performed between all prophages and CRISPR-spacer sequences. We established five different matching thresholds to explore potential interactions: (a) identity = 100%, coverage = 100% (Camarillo-Guerrero et al., 2021; Pei et al., 2024); (b) coverage = 100%, 0–2 mismatches (Kieft et al., 2021); (c) coverage = 95%, 0–1 mismatch (Nayfach et al., 2021); (d) identity = 95%, coverage >95% (Benler et al., 2021); (e) identity = 80%, coverage = 90%, 0–2 mismatches (Johansen et al., 2023); and (f) identity >90%, coverage >75% (Yan et al., 2023).

### 2.4 Defense systems prediction for all prokaryotic hosts and prophages

We first utilized CRISPRCasFinder (Couvin et al., 2018) (v4.3.2) with default parameters to predict CRISPR-Cas systems in prokaryotic hosts and prophages containing spacers predicted by MinCED, resulting in the identification of 240 CRISPR-Cas systems. Furthermore, we used DefenseFinder with default parameters to predict other defense systems for all 7,524 prokaryotic hosts and 10,742 identified prophages, and a total of 10,448 other defense systems were identified.

### 2.5 Genetic codes assessment and functional gene annotation for all identified prophages

Prodigal (Hyatt et al., 2010) (v2.50) was used to identify open reading frames (ORFs) of 10,742 prophage genomes under the standard genetic code (code 11) and three alternative genetic codes: TAG recoding (code 15), TAA recoding (code 90) and TGA recording (code 91) as described by Nayfach et al. (2021). Briefly, for a prophage with a genome size <100 kb, if its protein-coding density with the genetic codes 15, 90, or 91 increased >10% compared to that with the standard genetic code 11, we considered that this prophage genome tended to use the corresponding alternative genetic code. For those prophages with a genome size ≥100 kb, the threshold for considering the utilization of alternative genetic code was the increase of protein-coding density >5%.

In total, 311,891 protein-coding genes were identified from 10,742 prophage genomes using Prodigal with alternative genetic codes, and genes were annotated based on HMM searches against the Pfam-A (Mistry et al., 2021), IGRFAM (Haft et al., 2003), and VOGDB^1^ protein family databases. All searches were performed using the hmmsearch utility in the HMMER package (v.3.1b2) (Potter et al., 2018) with the “-E 1e-5” option, and each gene was annotated by each database according to its top-scoring alignment. Furthermore, to identify integrase and tail fiber proteins from prophage protein-coding genes, we first collected integrase and tail fiber proteins of DNA viruses from the NR database. We utilized Diamond (blastp) (Buchfink et al., 2015) to search the constructed integrase and tail fiber protein database for prophage protein-coding genes with the option “--more-sensitive -e 1e-5.” Additionally, the phylogenetic trees were constructed using identified integrase and tail fiber proteins from all prophages. Briefly, these integrase and tail fiber proteins were generated alignment sequences using MAFFT (v7.490) (Katoh and Standley, 2013), and these alignment sequences were trimmed using trimAl (v1.4.rev22) (Capella-Gutierrez et al., 2009). The phylogenetic trees were finally generated using FastTreeMP (v2.1.10) (Price et al., 2010) and visualized and annotated using iToL^2^ (Letunic and Bork, 2016).

### 2.6 Prediction of AMGs, ARGs, and VFGs among prophage genomes

Prophage-encoded auxiliary metabolic genes (AMGs) were annotated using the VIBRANT (v1.2.1) (Kieft et al., 2020) and DRAM-v (v1.3.5) (Shaffer et al., 2020). Briefly, proteins encoded by phage genomes were first scored by VirSorter2 (v2.2.2), and then, the scored proteins were annotated using DRAM-v with default options. AMGs were also annotated using the VIBRANT and assigned to the metabolic pathways using the KEGG database. Only those AMGs annotated by both the VIBRANT and DRAM-v were retained for further analyses. We used the ColabFold, which combined the fast homology search by MMseqs2 (v2.0) (Steinegger and Soding, 2017) with the AlphaFold2 (v2.0) (Tunyasuvunakool et al., 2021) to predict three-dimensional structures of AMGs. Five models in the AlphaFold2 were generated for each protein, and the highest-ranked model was used for structural alignments. Visualization, superimposition, and RMSD value calculation were performed using ChimeraX (v1.7) (Pettersen et al., 2021) with default parameters.

The proteins of all prophage genomes were compared to of the Comprehensive Antibiotic Resistance Database (CARD) using the Resistance Gene Identifier [RGI (Alcock et al., 2020), v. 5.1.0] to identify potential ARGs (the strict model with default parameters) and were aligned with the Virulence Factors Database (VFDB)^3^ using BLASTp to identify putative virulence factors with a threshold of identity ≥30% and coverage ≥70%.

### 2.7 Clustering of prophages and phylogenetic trees construction

To evaluate the novelty of porcine gut-derived prophages, we first collected putative viral genomes from a large porcine gut virome study [PVD (Mi et al., 2024)]. To exclude the effect of genome fragmentation, we used MH prophages (this study) and MH viral genomes (PVD) to cluster into the species-level viral clusters, the genus-level viral clusters, and the family-level viral clusters as described by Nayfach et al. (2021). We further incorporated viral genomes from three large human gut virome studies [MGV (Nayfach et al., 2021), GPD (Camarillo-Guerrero et al., 2021), and GVD (Gregory et al., 2020)] to form species-level clustering.

Furthermore, we constructed the phylogenetic trees of Caudoviricetes and crAss-like phages. Briefly, we first identified a set of 77 gene markers (Nayfach et al., 2021) of Caudoviricetes genomes from the predicted protein sequences based on individually searching against HMM profiles for the 77 markers using HMMER. We then trimmed and concatenated individual marker alignments to retain those genome fragments with less than 50% gaps using trimAl (v1.4.rev22) (Capella-Gutierrez et al., 2009). We only kept those viral genomes containing at least three markers and existing in >5% of alignment columns. Finally, the phylogenetic tree was constructed using the bootstrap generated using FastTreeMP and visualized using iTOL (see text footnote 2). To construct the phylogenetic trees of crAss-like phages based on the large terminase subunit (TerL) structural proteins, BLASTP and HMMER searches were performed against a custom structural protein database. And then, the resulting protein sequences were subsequently trimmed, aligned, and used for phylogenetic tree construction following the methods described above.

### 2.8 Data visualization and statistical analysis

All statistical analyses and data visualization were performed using the packages in R (v4.2.1).

## 3 Results

### 3.1 Comprehensive identification of prophages harbored in pig gut prokaryotes

**Figure 1:**
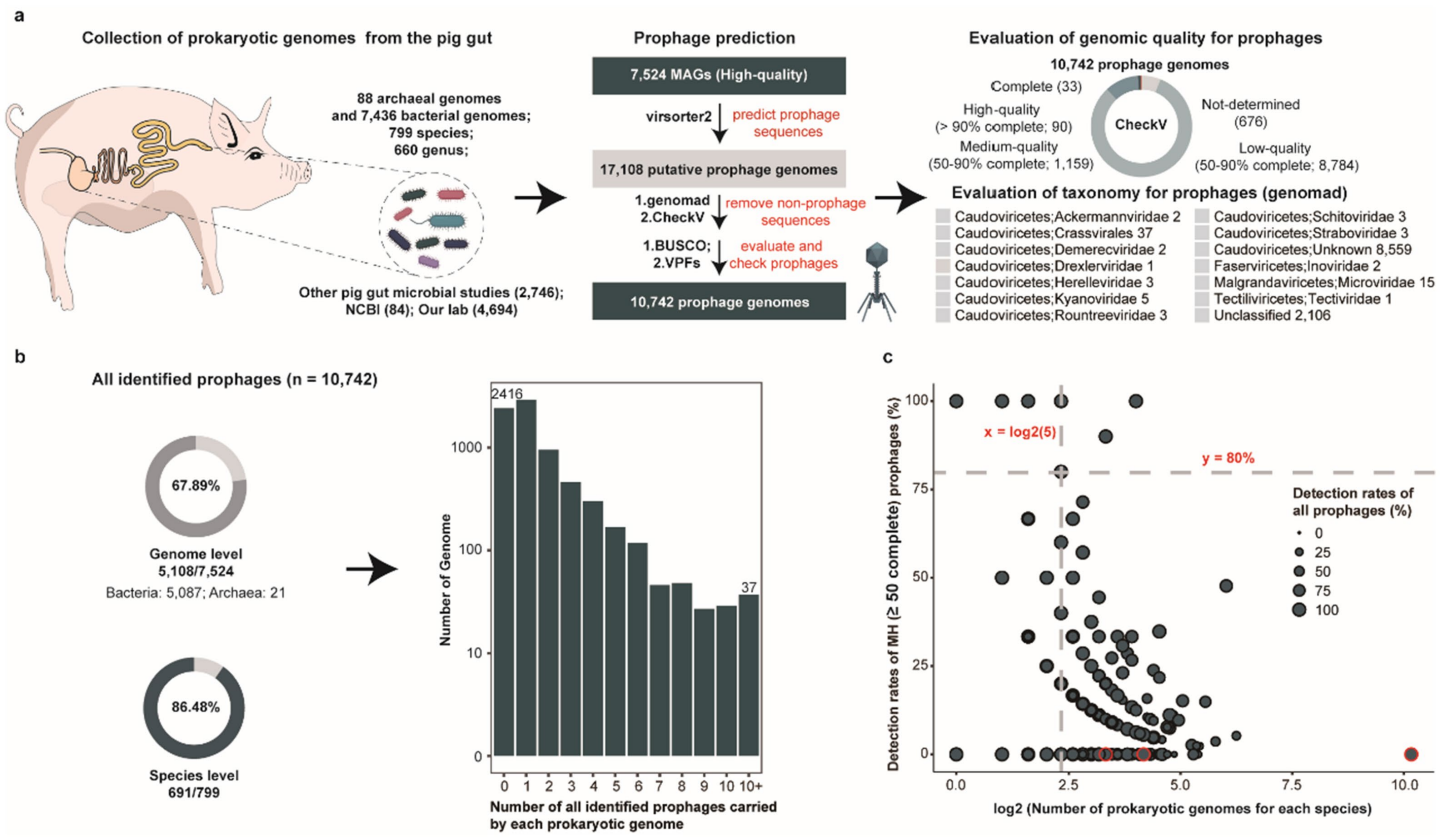
Overview of porcine gut-derived prokaryotic prophages. **(a)** Overview of the pipeline used for identifying porcine gut-derived prokaryotic prophages, including bacterial and archaeal genomes collection, prophage prediction, genomic completeness assessment, and taxonomic classification of prophages. **(b)** The proportion of all identified prophages at the genome level and the species level (pie charts), along with the distribution of the number of prophages per prokaryotic genome (bar chart). **(c)** The proportion of all identified prophages and MH prophages for each bacterial and archaeal species genome. The dots represent different bacterial and archaeal species.

To systematically investigate the characteristics and distribution of prophages within the porcine intestinal microbiome, we screened 7,524 prokaryotic metagenome-assembled genomes (MAGs, comprising 84 genomes from the NCBI, 2,746 from three other published studies about pig gut microorganisms, and 4,694 generated in our laboratory) derived from metagenomic sequencing data of swine gut microbiota (Supplementary Table S1), representing 12 phyla, 20 classes, 34 orders, 65 families, 188 genera, and 439 species (Figure 1a). Using a customized prophage identification pipeline, we identified 10,742 prophages with genome size from 5 kbp to 555.978 kbp and median size: 24.09 kbp (Supplementary Table S2), among which 1,282 prophage genomes exhibited medium-to-high quality (≥50% completeness) while 8,636 prophage genomes could be taxonomically classified, the vast majority were restricted to higher ranks, with only 40 confidently assigned at the family level and were primarily annotated as the members of known or unclassified viral families within the class Caudoviricetes, highlighting that a large number of potential new prophages remain to be characterized. Notably, these prophages were identified in 67.89% (5,108/7524) of prokaryotic genomes, encompassing 86.48% (691/799) of bacterial and archaeal species (Figure 1b). Striking variation in the numbers of prophages carried by each prokaryotic genome was observed across prokaryotes, with 2,917 genomes harboring a single prophage while 37 genomes contained more than 10 prophages.

Given the presence of incomplete phage fragments among the predicted prophage genomes, we established a subset catalogue comprising 1,282 medium-to-high quality (MH) prophage genomes. These 1,282 prophage genomes were distributed across 12.39% (932/7,524) of prokaryotic genomes and 40.43% (323/799) of prokaryotic species (Supplementary Figure S1a). However, 204 prokaryotic genomes harbored multiple medium-to-high quality prophages, which further underscores the highly uneven distribution across porcine intestinal prokaryotic genomes. Furthermore, to explore the relationship between prophage distribution and genomic GC content, we calculated the GC content of each prokaryotic genome carried prophage genome. While the majority of bacterial genomes exhibited GC contents ranging from 40 to 60%, archaeal genomes predominantly showed 20–40% GC contents (Supplementary Figure S1b). We focused on the distribution of prophages across different genomes in the same species. A total of 691 prokaryotic species were identified prophages in their genomes, with four species having ≥80% prokaryotic genomes identified prophages (Figure 1c). Notably, all these four species were conditionally pathogenic bacteria in the humans including *Escherichia fergusonii* (100%, 16/16), *Citrobacter portucalensis* (100%, 5/5), *Klebsiella pneumoniae* (90%, 9/10), and *Parabacteroides distasonis* (80%, 4/5), demonstrating significantly higher occurrence than other species. Intriguingly, several bacterial species, such as *CAG-317 sp000433215* (0%, 0/1,149), *UBA644 sp002299265* (0%, 0/18), and *Ruminiclostridium_E* sp016297165 (0%, 0/10), did not have any medium-to-high quality prophages detected in their genomes, but incomplete prophage genome fragments were observed in their genomes (Supplementary Table S3). Overall, substantial heterogeneity in prophage distribution was observed across different prokaryotic genomes in the porcine gut, and compared with symbiotic bacteria, specific pathogenic bacteria were more likely to carry prophages.

### 3.2 CRISPR spacer matching analysis reveals the potential prokaryotic host range and inter-prophage interactions

**Figure 2:**
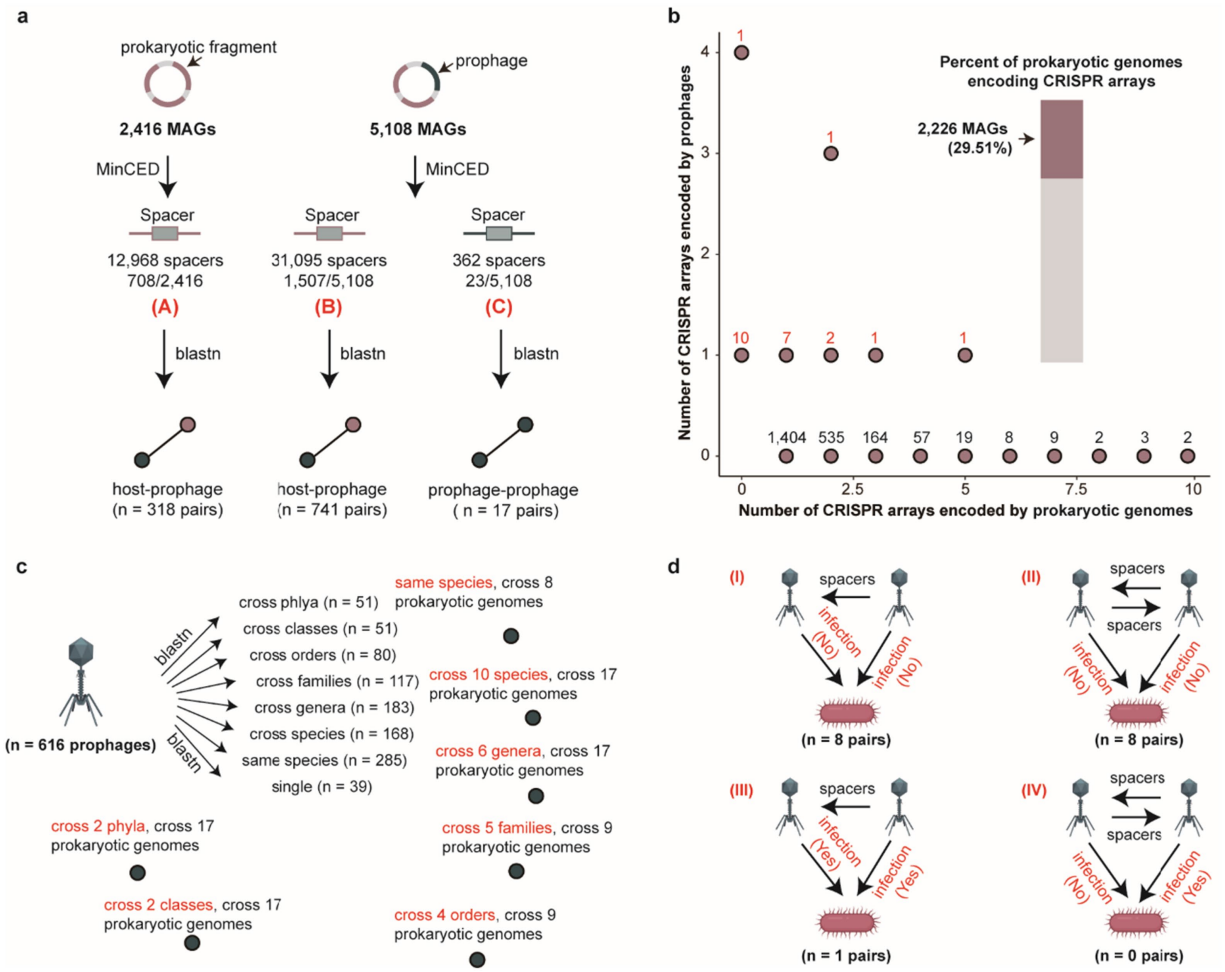
Host range of porcine gut-derived bacterial and archaeal prophages and patterns of inter-prophage interactions. **(a)** Schematic overview of CRISPR spacer matching analysis for prophages and hosts. **(b)** The number of CRISPR arrays encoded by prokaryotic genomes (*x*-axis) and prophage (*y*-axis) genomes, and the percent of prokaryotic genomes encoding CRISPR arrays. **(c)** The distribution of infection host range for prophages by CRISPR spacer matching. **(d)** The distribution of inter-prophage interaction types with CRISPR spacer matching.

Prophages typically alternate between lysogenic and lytic cycles, reflecting dynamic infection models (Touchon et al., 2016). Understanding the potential for horizontal transmission of prophages across distinct bacterial and archaeal hosts is crucial for unraveling the complex tripartite interactions among phages, prokaryotes, and their host organisms (Zeng et al., 2016; Yin et al., 2016). CRISPR spacer sequences provide a powerful tool for reconstructing the historical infection events of phage (Medvedeva et al., 2022; Wu et al., 2024). We identified a total 44,425 spacer sequences from 29.44% (2,215/7,524) prokaryotic genomes and 23 prophage genomes, and after matching with different thresholds for spacer matching, we obtained different specific numbers of relationships. Specially, we obtained 1,059 host-prophage pairs and 17 prophage-prophage interactions, 2,712 host-prophage pairs and 25 prophage-prophage interactions, 3,346 host-prophage pairs and 29 prophage-prophage interactions, 3,350 host-prophage pairs and 29 prophage-prophage interactions, 8,583 host-prophage pairs and 67 prophage-prophage interactions, and 25,752 host-prophage pairs and 230 prophage-prophage interactions using five distinct sets of parameter settings. Considering the importance of interaction accuracy, we retained the most stringent threshold (100% identity and 100% coverage) for our final analysis (Figure 2a and Supplementary Tables S4, S5). Notably, the majority of prokaryotic genomes encoded a maximum of two CRISPR arrays, whereas most prophages contained at most one CRISPR array (Figure 2b). CRISPR spacer targeting analysis further revealed 616 prophages with putative prokaryotic hosts. Among all spacer-targeted prophages, 70.29% (433/616) exhibited high host specificity (specialist phages, targeting a single bacterial/archaeal genus), while the 29.71% (183/616) demonstrated broad host ranges (generalist phages, targeting 2–6 distinct bacterial/archaeal genera) (Supplementary Figure S1d). Notably, a subset of prophages (*n* = 51) displayed cross-phylum infection capability, indicating an exceptional potential for broad-host-range infectivity (Figure 2c). Subsequent analysis of prophage-prophage interactions revealed three principal interaction modes (I, II, and III) (Figure 2d). Considering both bacterial host genomes and prophages were predicted to contain spacer sequences, three interaction patterns naturally emerged: (I) a prophage with a predicted spacer matches another prophage genome; (II) both prophages contain predicted spacers that match each other; (III) a prophage matches both its bacterial host and another prophage, with the two prophages sharing a common host. Intriguingly, we observed that the majority of prophage-prophage interactions predominantly adopted modes I and II, suggesting that during integration into prokaryotic host genomes, phages may preferentially capture and incorporate sequences from invading phage sequences into their spacer arrays, potentially establishing a phage-mediated immune-like defense mechanism analogous to host CRISPR systems (Figure 2d).

### 3.3 Prophages augment or confer defense mechanisms in prokaryotic hosts against exogenous phage predation

**Figure 3:**
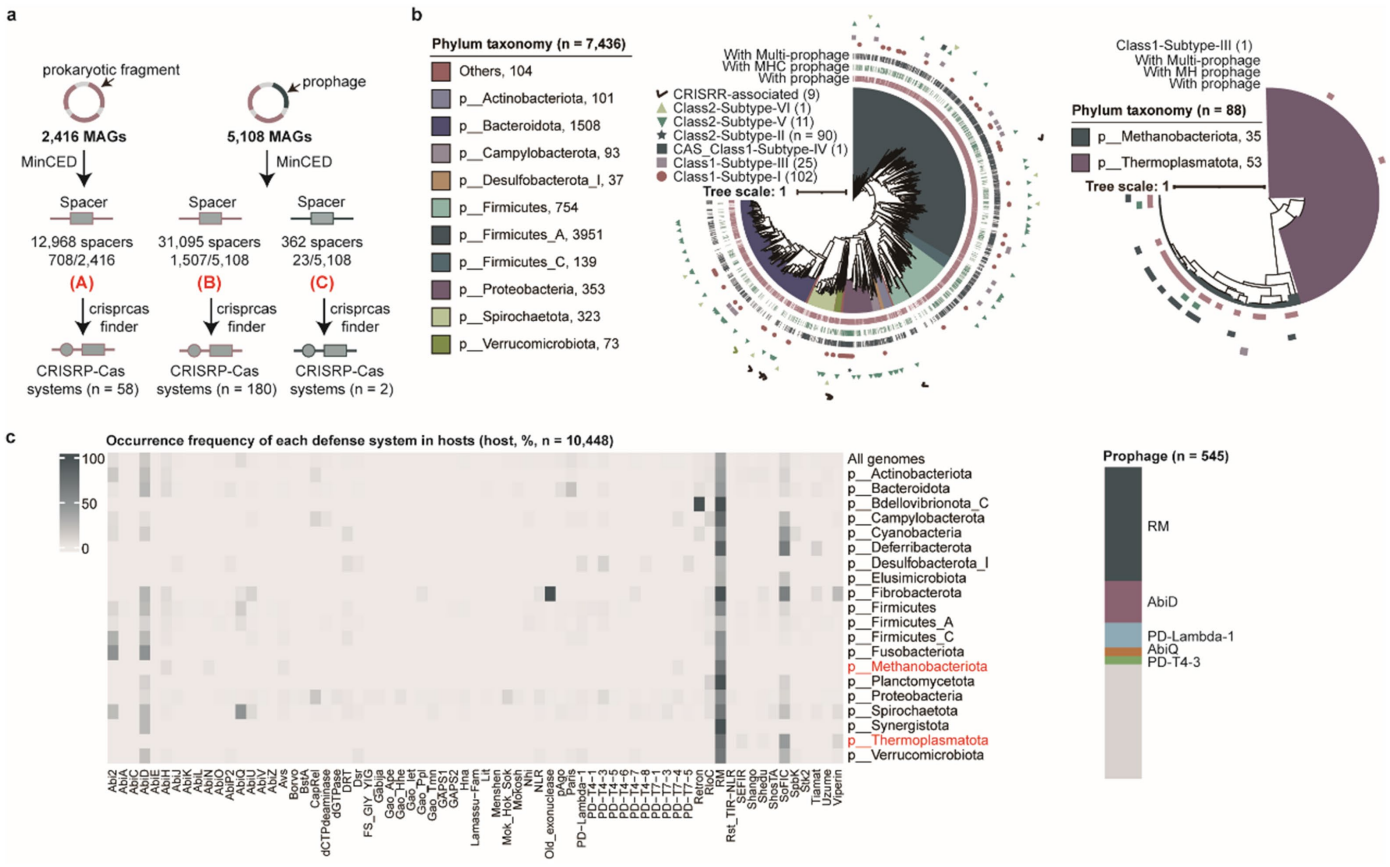
Characterization of defense mechanisms in prokaryotic hosts and prophages. **(a)** Overview of the pipeline used to identify CRISPR-Cas systems in prokaryotic hosts and prophages. **(b)** The phylogenetic tree analysis of CRISPR-Cas systems and associated prophage types in bacterial (left) and archaeal (right) genomes. Different clades correspond to distinct bacterial and archaeal phyla. The outer rings represent CRISPR-Cas system types and prophage types. **(c)** The distribution of other antiviral defense systems (excluding CRISPR-Cas systems) in prokaryotic hosts and prophages. The heatmap (left) shows the frequency of different defense systems across host genomes at the phylum level and all genomes, with archaeal phyla labeled in red. The stacked diagram (right) illustrates the proportion of other defense systems identified in all prophage genomes.

Host microorganisms deploy diverse defense mechanisms, including CRISPR-Cas, restriction-modification (RM), and abortive infection (Abi) systems to counteract phage invasion (Bernheim and Sorek, 2020; Makarova et al., 2020; Jurėnas et al., 2022). Intriguingly, certain archaeal viruses and huge phages have been reported to encode CRISPR-Cas systems for eliminating competing phages (Al-Shayeb et al., 2020; Wu et al., 2024). We successfully predicted CRISPR-Cas systems in prokaryotic genomes (*n* = 238) and prophages (*n* = 2) using CRISPRCasFinder (Figure 3a and Supplementary Table S6). Although CRISPR-Cas systems were identified in both prokaryotic genomes and prophages, their abundance was significantly lower than the number of CRISPR arrays detected in prokaryotic genomes (*n* = 2,215) and prophages (*n* = 23), suggesting that the majority of CRISPR arrays exist in isolation and likely cannot mediate functional CRISPR-Cas-mediated host immunity. However, this interpretation may be influenced by the incomplete assembly of both the prokaryotic host and prophage genomes. Subsequent characterization of CRISPR-Cas systems encoded in prokaryotic genomes revealed their classification into two classes, four types, and eleven subtypes, with Class 1-Subtype I (*n* = 103) and Class 2-Subtype II (*n* = 90) representing the predominant CRISPR-Cas system types among prokaryotic genomes (Figure 3b). Concomitantly, we surprisingly observed that CRISPR-Cas systems encoded by prophages themselves were incomplete, lacking core Cas effector proteins such as Cas3, Cas9, or Cas12. However, these prophages could functionally leverage host-derived Cas effectors (e.g., Cas9) from their prokaryotic hosts to execute CRISPR-Cas-mediated cleavage of foreign DNA (Supplementary Figure S2a), thereby enhancing the host’s antiviral defense against competing phage infections.

In addition to CRISPR-Cas systems, prokaryotic genomes have evolved a multitude of antiviral mechanisms in their evolutionary arms race against viral pathogens (Chopin et al., 2005). We systematically identified 10,448 antiviral defense systems (including those encoded within prophages) across 7,524 prokaryotic genomes beyond CRISPR-Cas systems. Among these, 38.40% (2,889/7,524) of prokaryotic hosts predominantly relied on restriction-modification (RM) systems, while 15.23% (1,146/7,524) utilized AbiD systems as their main defense strategy. Notably, 5.07% (545/10,742) of the 10,742 prophages encoded defense systems that could potentially contribute to their host protections. After stringent filtering to exclude potential contamination from host-derived sequences, this finding suggests that prophages may acquire antiviral defense systems via horizontal gene transfer (HGT) to enhance host defense capacity against competing phages (Figure 3c).

### 3.4 Functional insights into prophages in the porcine gut microbiota

**Figure 4:**
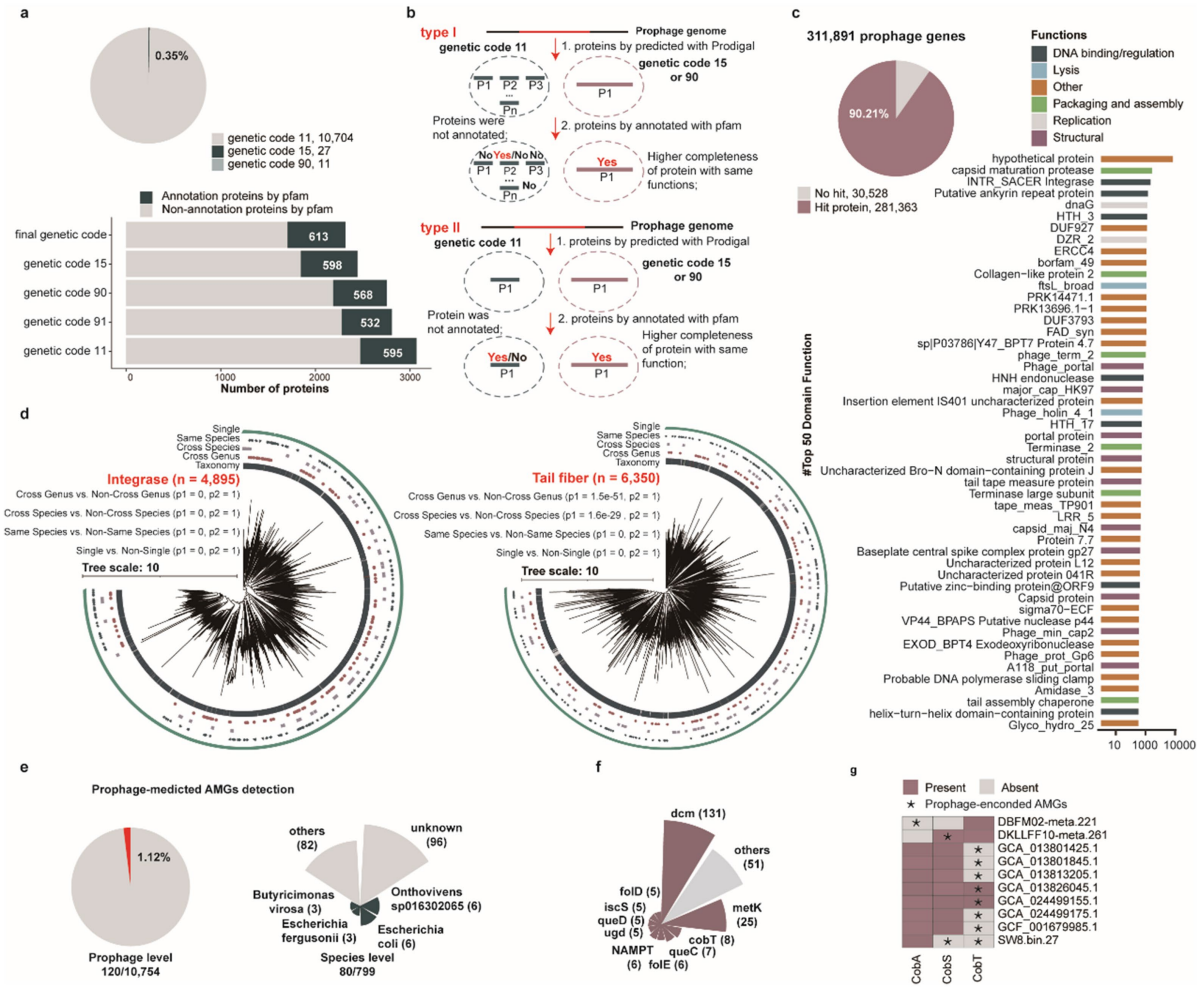
Functional characterization of porcine gut-derived prophages. **(a)** The proportion of using alternative genetic codes for all identified prophages (pie chart), the number of proteins annotated by the Pfam-A database for prophages using five alternative genetic codes. **(b)** Comparative analysis demonstrating that alternative genetic code usage results in fewer total proteins compared using the standard genetic code but a greater number of annotated proteins compared to predictions using the standard genetic code. **(c)** The proportion of annotated proteins (pie chart) and the distribution of top 50 function for all identified prophages (bar chart). **(d)** The phylogenetic tree analysis of integrase proteins and tail fiber proteins encoded by all identified prophages. **(e)** The proportion of prophages encoding AMGs, and the distribution of prophages with AMGs in prokaryotic genomes at the species level. **(f)** The distribution of AMGs encoded by all identified prophages. **(g)** The present and absent analysis of vitamin B12-related AMGs for prokaryotic genomes.

Several phages, including huge phages and crAss-like phages, have been demonstrated to employ alternative codon recoding strategies (Devoto et al., 2019; Yutin et al., 2021; Lou et al., 2024), which play crucial roles in regulating viral replication and gene expression mechanisms. To investigate the prevalence of alternative codon usage patterns among porcine intestinal prophages, we performed protein prediction using four genetic codes (genetic code 11: standard code, genetic code 15, genetic code 90, and genetic code 91), optimizing genetic code selection based on total alignment scores. Our analysis revealed that 0.35% (38/10,742) prophages utilize alternative codon strategies for protein encoding. Comparative Pfam annotation of these alternatively recoded proteins demonstrated that, although fewer proteins were predicted under alternative codon usage (2,317 vs. 3,068), a higher number of Pfam annotations (613 vs. 595) were achieved compared to standard codon-derived predictions (Figure 4a). Comparative analysis further demonstrated that, while the majority of protein predictions overlapped between the two strategies, alternative codon usage enabled the assembly of previously fragmented protein predictions into cohesive, full-length polypeptides, often yielding larger and more complete protein architectures than standard code predictions (Figure 4b). This finding provides a mechanistic explanation for the enhanced functional annotation yields observed with alternative codon recoding.

To further investigate the functional potential of prophages within the porcine intestinal microbiome, we analyzed 311,891 proteins encoded by 10,742 prophages against hidden Markov model (HMM) databases including TIGRFAM, Pfam, and VOGDB. Collectively, 9.79% (30,534/311,891) of prophage genes exhibited no significant matches in any database (Supplementary Table S7). The majority of annotated genes were classified as hypothetical proteins or lacked assigned biological functions, highlighting the limited functional characterization of porcine intestinal prophages in current genomic databases. Concurrently, several prophage-encoded proteins exhibited canonical viral functionalities, including capsid formation, packaging, lysis, lysozymes, and transcriptional regulation (Figure 4c), spanning genes associated with core phage functional modules. Notably, several prophage-encoded proteins were annotated as helix-turn-helix (HTH) motifs, which primarily mediate bacterial chromosomal binding, a molecular mechanism that may constitute a critical factor facilitating phage integration into bacterial genomes. Intriguingly, a subset of prophage-encoded proteins was annotated as HNH endonuclease domains, which may facilitate targeted cleavage of specific DNA sequences derived from competing phages (Bellas et al., 2020), suggesting a putative defense mechanism against rival viral elements. Similarly, diverse glycosyltransferases and methyltransferases were annotated, which may enable viruses to evade host defense systems (Markine-Goriaynoff et al., 2004; Jeudy et al., 2020).

Integrase, a key enzyme mediating site-specific recombination and facilitating the stable integration of phage genomes into bacterial/archaeal host chromosome (Howard-Varona et al., 2017), was also extensively characterized. Similarly, phage tail-fiber proteins constitute critical structural components of the viral tail apparatus, directly mediating host recognition, adhesion, and infection initiation (Yehl et al., 2019; Patel et al., 2024), were indicated. These proteins represent central molecular determinants governing phage host specificity and infection efficiency. To investigate whether integrase and tail tube proteins contribute to prophage host range, we constructed phylogenetic trees of these proteins to evaluate their host distribution patterns. Phylogenetic analysis revealed that evolutionary distances were significantly smaller within groups infecting similar hosts (single, same species, cross species, and cross genus) compared to between-group distances (Figure 4d, *p* < 0.05). This finding suggests that prophage-encoded integrase and tail tube proteins may be crucial determinants of phage host specificity.

Prophage-encoded auxiliary metabolic genes (AMGs) can modulate host metabolism, thereby enhancing or reprogramming metabolic pathways (Yi et al., 2023). Subsequent systematic annotation of AMGs across all prophage genomes revealed that 1.12% (120/10,754) of prophages harbored AMGs, originating from 10.01% (80/799) of host species (Figure 4e and Supplementary Table S8). Among these auxiliary metabolic genes (AMGs), the *dcm* gene was the most prevalent, followed by *metK* and *cobT* genes (Figure 4f). Notably, the *cobT* gene, a critical determinant of vitamin B12 biosynthesis, is involved in the production of an essential nutrient that must be acquired exogenously through the diet or, to a limited extent, synthesized endogenously by the gut microbiota in swine and humans (Wienhausen et al., 2024). Therefore, we conducted an in-depth investigation of prophage-associated AMGs involved in vitamin B12 biosynthesis, focusing on *cobA*, *cobS*, and *cobT* genes. Our findings suggest that prophage-mediated transfer of these genes may supplement or enhance host biosynthetic capabilities, providing selective advantages through enhanced nutritional biosynthesis (Figure 4g). Furthermore, to delineate the metabolic augmentation potential of prophage-encoded AMGs (*cobA*, *cobS*, and *cobT*), we performed comparative analyses of prophage-encoded AMGs and their bacterial/archaeal host homologs, including protein identity (PI) assessments (Supplementary Figure S2b) and three-dimensional structural comparisons (Supplementary Figure S2c). These investigations demonstrated functional equivalence between prophage-derived AMGs and native host-encoded genes. This molecular convergence strongly supports the hypothesis that prophage integration supplements or amplifies host metabolic networks (Supplementary Figure S2d), ultimately influencing microbial physiological functionality through auxiliary biosynthetic pathway modulation. Furthermore, we assessed the potential functionality of prophages based on the integrity of their genomic features. Our analysis revealed that approximately 997 genomes in the MHC harbor lysis-related genes, and 263 genomes contain integration-related genes. The presence of these functional modules is likely to facilitate the completion of the prophage life cycle and enable them to exert their biological functions (Supplementary Table S9).

### 3.5 Prophage-mediated mobilization of antibiotic resistance genes and diverse virulence factor genes in the porcine gut microbiota

**Figure 5:**
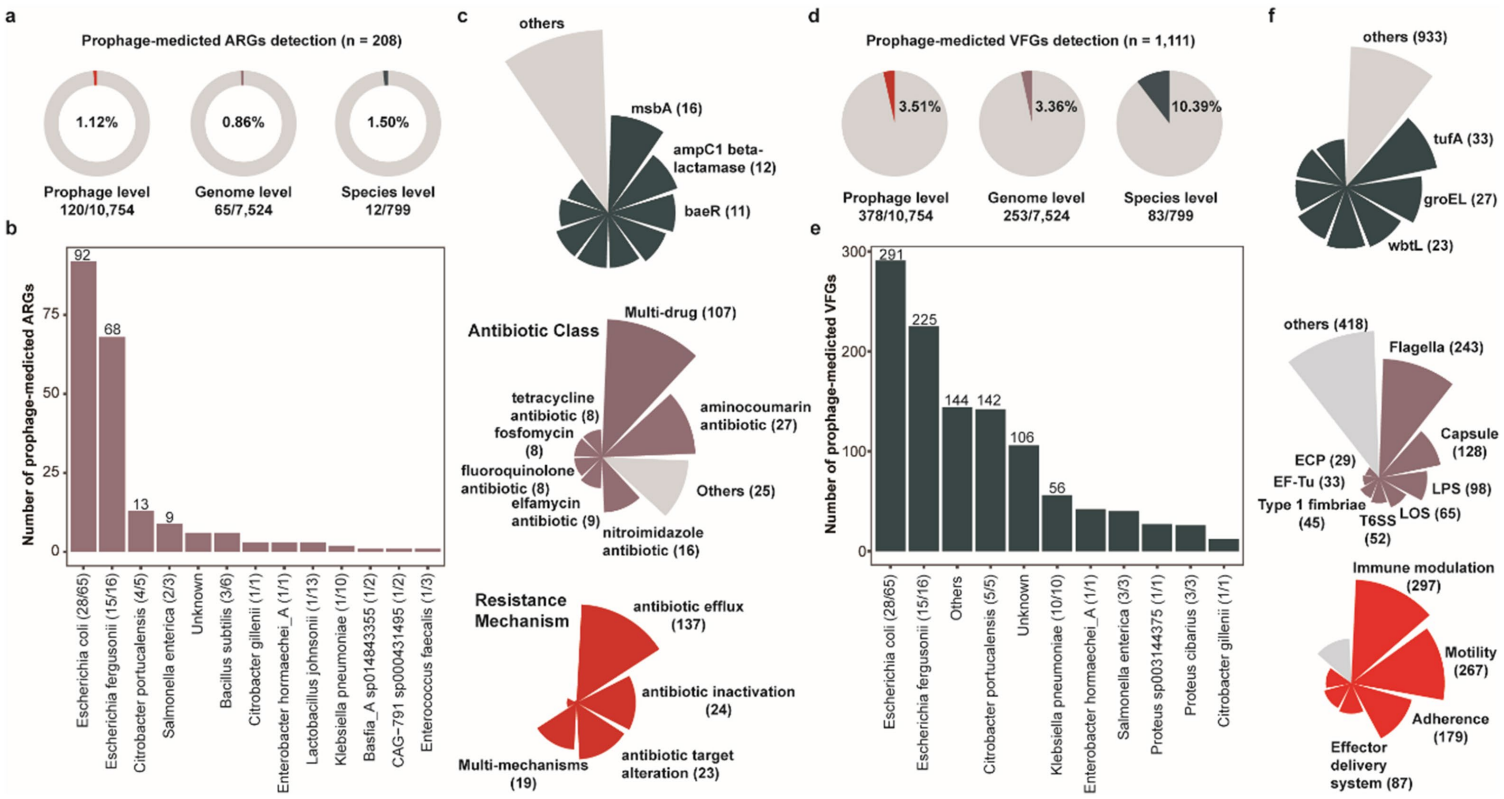
The antibiotic resistance genes and diverse virulence factor genes encoded by porcine gut-derived prophages. **(a)** The proportion of prophage-mediated ARGs at the prophage, the host genome, and the host genus level. **(b)** The number of prophage-mediated ARGs for the host species. **(c)** The distribution of prophage-mediated ARGs, including the antibiotic class and resistance mechanism. **(d)** The proportion of prophage-mediated VFGs at the prophage, the host genome, and the host genus level. **(e)** The number of prophage-mediated VFGs for the host species. **(f)** The distribution of prophage-mediated VFGs, including the antibiotic class and resistance mechanism.

Prophages serve as pivotal vectors for horizontal gene transfer (HGT), facilitating not only the dissemination of antibiotic resistance genes (ARGs) but also enhancing host pathogenicity through the transfer of virulence factor genes (VFGs) to bacterial hosts (Pei et al., 2024), thereby driving the evolution of bacterial virulence. To comprehensively characterize and assess the transmission risks of ARGs and VFGs mediated by prophages within porcine intestinal bacterial/archaeal hosts, we conducted a systematic genomic analysis of all 10,742 prophage genomes. Specifically, we identified 208 putative ARGs across 120 prophages, associated with 65 bacterial/archaeal genomes (0.86%, 65/7,524) and 12 prokaryotic species (1.50%, 12/799) (Figure 5a and Supplementary Table S10). Notably, *Escherichia coli* harbored the highest number of prophage-associated ARGs (*n* = 92), with 43.08% (28/65) of *E. coli* strains carrying ARG-bearing prophages. This was followed by *Escherichia fergusonii* (*n* = 68 ARGs, 15 of 16 strains), *Citrobacter portucalensis* (*n* = 13 ARGs, 4 of 5 strains), and *Salmonella enterica* (*n* = 9 ARGs, 2 of 3 strains) (Figure 5b). Among the identified ARGs, multidrug resistance (n = 107), aminocoumarin resistance (*n* = 27), and nitroimidazole resistance (*n* = 16) were the most prevalent categories, followed by elfamycin resistance (*n* = 9), fluoroquinolone resistance (*n* = 8), fosfomycin resistance (*n* = 8), and tetracycline resistance (*n* = 8) (Figure 5c). Primary antibiotic resistance mechanisms observed in porcine intestinal bacterial prophages encompassed antibiotic efflux, antibiotic inactivation, antibiotic target alteration, and multi-mechanism resistance. Furthermore, our analysis revealed that cross-species prophages exhibited a higher ARG detection frequency (3.70%, 3/81) compared to prophages with restricted host ranges. However, single-host lineages (Single) displayed greater diversity in their ARG repertoires relative to those with broad host ranges (cross genera, cross species, and same species) (Supplementary Figure S2e). Overall, the presence of ARGs within cross-species prophages underscores their enhanced potential for mediating the dissemination of resistance determinants across taxonomic boundaries.

Similarly, we detected 1,111 putative virulence factor genes (VFGs) across 378 prophage genomes, associated with 253 bacterial/archaeal genomes (3.36%, 253/7,524) and 83 prokaryotic species (10.39%, 83/799) (Figure 5d and Supplementary Table S11). Notably, *Escherichia coli* harbored the highest number of prophage-associated virulence factor genes (VFGs) (*n* = 291), with 43.08% (28/65) of *E. coli* strains carrying VFG-bearing prophages. This was followed by *Escherichia fergusonii* (*n* = 225 VFGs, 15 of 16 strains), *Citrobacter portucalensis* (*n* = 142 VFGs, 5 of 5 strains), and *Klebsiella pneumoniae* (*n* = 56 VFGs, 10 of 10 strains) (Figure 5e). Among these virulence factors, Flagella (*n* = 243), Capsule (*n* = 128), and Lipopolysaccharide (LPS, *n* = 98) were the most prevalent, followed by Lipooligosaccharide (LOS, *n* = 65), Type VI Secretion System (T6SS, *n* = 52), and Type 1 fimbriae (*n* = 45 and Figure 5f). Among all prophages harboring toxin-related VFGs, those with cross-species integration exhibited a higher detection frequency (11.11%, 9/81) compared to other integration modes (Supplementary Figure S2f). Moreover, broad-host-range prophages (cross genera, cross species, and same species) played a pivotal role in the dissemination of toxin gene, highlighting their substantial risk potential in promoting the spread of pathogenic trait across microbial communities.

### 3.6 Mining prophages reveals a fraction of viral dark matter in the porcine gut

**Figure 6:**
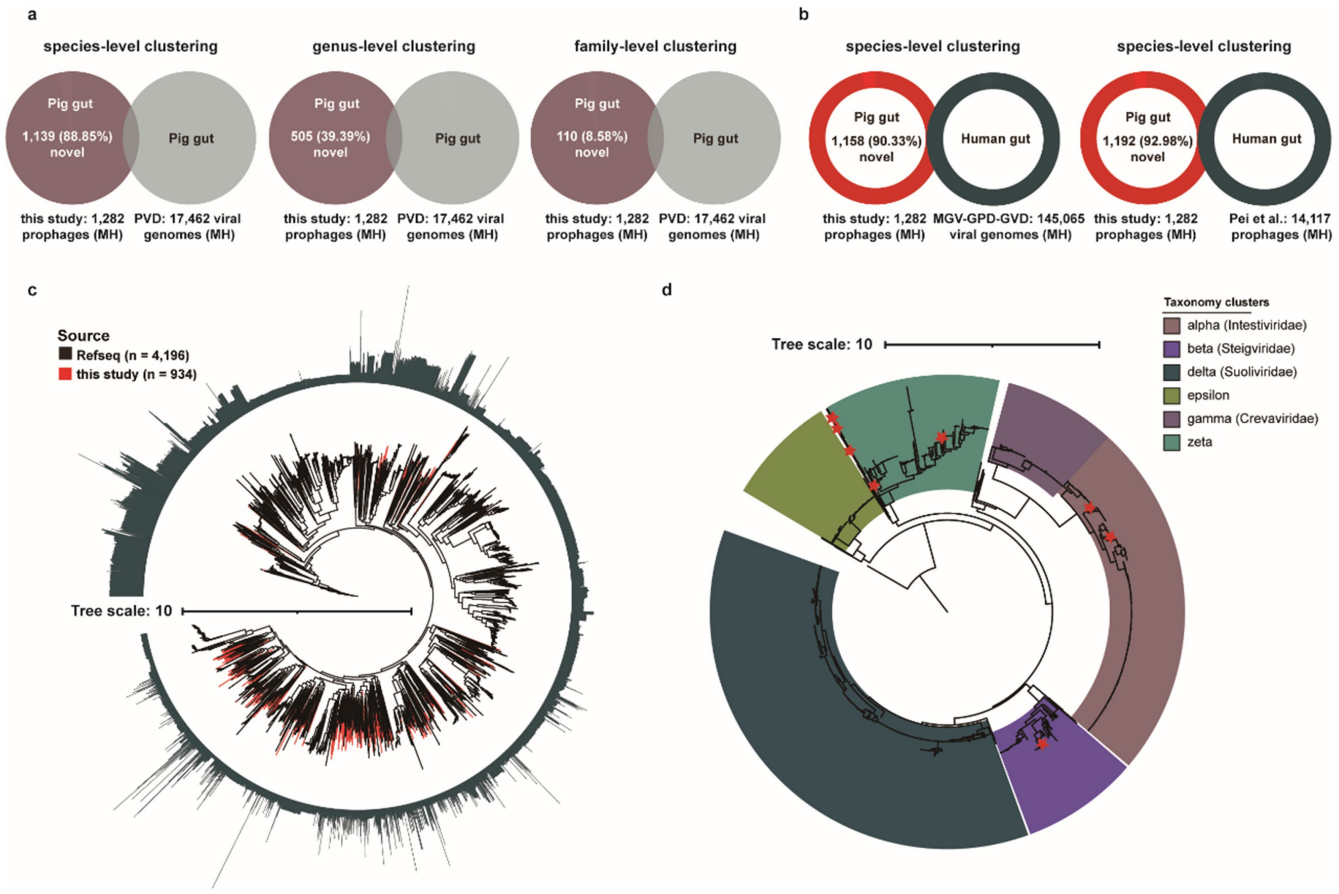
Characterization of the novel porcine gut-derived prophages. **(a)** The genome clustering analysis for MH prophages with previous large Porcine Virome Database (PVD) at the species-level clustering, the genus-level clustering, and the family-level clustering. **(b)** The genome clustering analysis for MH prophages with three major human gut virome datasets (MGV, GPD, and GVD) and human gut prophages (Pei et al., 2024) at the species-level. **(c)** The phylogenetic tree analysis of Caudoviricetes based on 77 marker genes for Caudoviricetes MH prophages and RefSeq’s Caudoviricetes sequences. The outer circle represents the length of genomes, and the red clades represent Caudoviricetes MH prophages in this study. **(d)** The phylogenetic tree analysis of crAss-like phages based on TerL proteins. The red stars represent TerL proteins of MH prophage in this study, and the color of clades represents the different crAss-like subfamilies.

Profiling of porcine intestinal virome can be performed either through bulk metagenomic sequencing or viral-like particle (VLP)-enriched sequencing to identify viral sequences. The recent release of a large-scale Porcine Virome Dataset (PVD) has significantly advanced research on swine gut-associated viral communities (Mi et al., 2024). To assess the contribution of our prophage dataset to existing porcine intestinal viral databases, we clustered all 1,282 medium-to-high (MH) quality prophage genomes against viral sequences in the PVD at species-, genus-, and family-level taxonomic resolutions (Figure 6a). Specifically, approximately 11.15% (143/1,282) of MH prophages exhibited sufficient species-level clustering matches with known porcine intestinal phages, while the remaining 88.85% (1,139/1,282) represent previously uncharacterized viral entities. Similarly, taxonomic clustering revealed 39.39% (505/1,282) and 8.85% (110/1,282) of MH prophages as previously uncharacterized viral entities at genus- and family-level clustering resolutions, respectively. These newly identified medium-to-high (MH) quality prophage genomes of bacterial/archaeal origin substantially expand the current reference genome databases for porcine gut phages. Subsequent comparative analysis against three large-scale human gut virome databases [Metagenomic Gut Virus catalogue (MGV), Gut Phage Database (GPD), and Gut Virome Database (GVD)] revealed that only 9.67% (124/1,282) of MH prophages formed sufficient species-level clustering matches with known human gut phages, whereas the vast majority, 90.33% (1,158/1,282), remained uncharacterized (Figure 6b). Certainly, compared with MH prophages from human guts, 92.98% (1,192/1,282) pig gut prophage genomes exhibited uniqueness (Figure 6b).

Furthermore, to evaluate the contribution of our dataset to the current Caudoviricetes class, we conducted a concatenated phylogenetic analysis of 934 MH prophage genomes classified within Caudoviricetes against 4,196 complete Caudoviricetes phage genomes from the RefSeq database, utilizing 77 conserved marker proteins (Figure 6c). Phylogenetic diversity analysis revealed that prophages from this study contributed 39.74% (867/2,182 PD units) of the total phylogenetic diversity (PD), substantially expanding the current diversity landscape of the Caudoviricetes class (Figure 6c).

CrAss-like phages, which dominate the human gut virome, are known to modulate intestinal bacterial abundance and diversity through host-specific interactions (Guerin et al., 2018; Yutin et al., 2021), have been linked to host metabolism, immune regulation, and disease susceptibility, including established associations to obesity, inflammatory bowel disease (Jansen and Matthijnssens, 2023). They belong to the Crassvirales order (hereafter referred to as crassviruses). Notably, substantial populations of crAss-like phages have also been reported in the intestinal ecosystems of non-human animals, suggesting conserved ecological roles across mammalian hosts. Subsequently, we investigated the presence of crAss-like phages within porcine intestinal prophages and assessed their distribution across six subclusters (alpha, beta, delta, epsilon, gamma, and zeta). Notably, four subclusters—alpha, beta, delta, and gamma—have been formally classified by the International Committee on Taxonomy of Viruses (ICTV) as distinct families: *Intestiviridae*, *Steigviridae*, *Suoliviridae*, and *Crevaviridae*, respectively (Figure 6d). ICTV currently classifies crAss-like phages into only four official subfamilies (alpha, beta, gamma, delta), while epsilon and zeta have not yet been included in the official classification system. Although these two subfamilies have been identified as independent branches in phylogenetic analyses, they lack sufficient taxonomic evidence (such as representative virus isolates, host infection verification, or complete functional characteristics) to meet ICTV’s strict classification criteria. Collectively, we identified 12 MH crAss-like phages (0.94%, 12/1,282), predominantly distributed within the zeta subcluster, followed by the alpha and beta subclusters.

## 4 Discussion

Prophages play crucial roles in shaping the ecology and evolution of microbe populations, with important consequences for higher-order ecological interactions (Wendling, 2023). In this study, we systematically investigated the diversity of prophages in the pig gut and further characterized their host range, functional attributes, and interactions with bacterial or archaeal hosts through large-scale analysis of porcine gut microbiota genomes. The highly uneven distribution of prophages and their exceptionally broad host ranges reveal potentially diverse interaction modalities between phages and their prokaryotic hosts. Prophage-encoded defense systems, particularly their influence on the integrity of host CRISPR-Cas systems, play a critical role in helping hosts resist infection by other phages. Auxiliary metabolic genes suggest that prophages may protect host prokaryotes from phage predation while enhancing or modifying host metabolic capabilities, thereby increasing prokaryotic fitness. The identification of antibiotic resistance genes and virulence factors encoded by prophages with cross-host potential underscores their inherent risk in disseminating resistance determinants and virulence traits. These findings demonstrate that prophages integrated into host prokaryotes’ genomes enhance prokaryotic fitness through multiple mechanisms, providing deeper insights into the role prophages play within the complex pig gut microbiome.

Through CRISPR spacer matching analysis, we gained a clearer understanding of the host distribution of pig gut prophages. Our study demonstrates that prophages were identified in 67.89% (5,108/7,524) of prokaryotic host genomes analyzed, yet only 12.39% (932/7,524) of these hosts harbored MH-associated prophages. The high frequency of prophage distribution in host genomes further confirms that, in the gut environment, the benefits prophages provide to their hosts are as indispensable as in other environments during the bacteriophage-host interactions (Bosi and Mascagni, 2019; Middelboe et al., 2025). Furthermore, the distribution of integrated prophages exhibits pronounced heterogeneity across host species, with a higher prevalence of MH-associated prophages identified in potential opportunistic pathogens. This suggests that opportunistic pathogens may undergo more phage-mediated horizontal gene transfer events. Moreover, these multi-niche-adapted pathogens demonstrate enhanced prophage integration propensity, likely conferring survival advantages across diverse environments through phage-mediated genomic plasticity (Tan et al., 2020).

Furthermore, leveraging infection histories archived in host CRISPR systems, we delineated the potential host ranges of prophages (Liao et al., 2024). Notably, certain phages exhibited broad-spectrum infectivity, even demonstrating cross-phylum infection capabilities that transcend established taxonomic barriers. This also implies that certain prophages may have the potential to infect multiple hosts upon induction, possibly even extending across different bacterial phyla. Interestingly, our genomic functional characterization analysis also revealed that the host range of prophages is closely linked to integrase structure, although further evidence is needed to substantiate this association.

Prophage-encoded defense systems provide resistance to distant phages through diverse mechanisms, including modification of cell surface receptors (Uc-Mass et al., 2004), inhibition of DNA translocations (McGrath et al., 2002). Previous work highlighted such prophage-encoded defense systems participate in inter-viral competition (Dedrick et al., 2017). Here, we also discovered that certain prophages integrated into prokaryotic hosts enhance or directly provide host defense mechanisms, fortifying the host’s adaptive immune capabilities through phage-mediated genetic augmentation. Notably, within tripartite phage-phage-host interactions, we observed that prophages preferentially acquire foreign invasive phage sequences through CRISPR spacer integration mechanisms. Our findings still require further validation through induction and infection experiments to assess the extent to which prophages influence the integrity of host defense systems. Interestingly, Brenes and Laub (2025) recently demonstrated that *E. coli* prophages encode a diverse arsenal of defense systems that protect against temperate phage infection. Collectively, these findings demonstrate that while some phages engage in predatory lytic cycles, leaving detectable infection signatures across diverse prokaryotic hosts, others establish nuanced symbiotic relationships following genomic integration without inducing host lysis. Such non-lytic phages predominantly enhance host adaptive capabilities through mutualistic interactions, suggesting phage survival strategies are selectively optimized based on intrinsic viral properties and host-specific genomic constraints. Furthermore, our study confirms that during prolonged evolutionary arms races with prokaryotic hosts, phages develop specialized evasion strategies to circumvent host defense systems (Wu et al., 2024).

Prophage-mediated gene transfer is primarily known to occur through generalized, specialized, and lateral transduction (Fillol-Salom et al., 2021), accompanied by the transfer of a wide array of functional genes into host genomes. Here, we found that in addition to prophage-encoded integrases, tail tube proteins may also play a role in shaping phage host specificity through molecular recognition mechanisms that govern infection tropism. This is consistent with the description of targeting mechanisms of tailed bacteriophages reported by Nobrega et al. (2018). AMGs are found in both lytic and temperate phages, and they have the potential to be utilized at any stage of host infection, such as photosynthesis (Mann et al., 2003), sulfur oxidation (Mann et al., 2003). Through functional annotation of prophage-encoded AMGs, we demonstrated that prophages in the pig gut enhance or modify host metabolic capabilities, enabling host bacteria to more efficiently synthesize essential metabolites such as vitamin B12, which is critical for porcine physiology. However, the carriage of antibiotic resistance genes and virulence factors by prophages, coupled with their broad host range, underscores their inherent risk of disseminating these genetic determinants within the porcine gut microbiota. Of course, our study mainly focuses on the genomic distribution of auxiliary metabolic genes, antibiotic resistance genes and virulence factors, which may have the potential to be expressed. Beyond inferring the functionality of prophages based on the presence of genes essential for their basic lifestyle, we did not directly investigate prophage activity. Moreover, our study lacks experimental validation to determine whether these prophages are truly functional. We acknowledge this as a limitation of the present work.

Furthermore, to assess the novelty of the phage genomes we identified compared to those in current public databases, naturally, this is directly related to the limited availability of porcine gut phage databases and the substantial heterogeneity observed among viral communities in the pig gut (Hu et al., 2024b). Unfortunately, due to the limited availability of sample information, our ability to further explore the factors influencing the pig gut virome was restricted. Although porcine and human prophage genomes exhibit substantial differences, the host distribution patterns of pig gut prophages show notable similarities to those in humans. In particular, potential pathogens such as *Escherichia coli* and *Klebsiella pneumoniae* appear more likely to harbor prophages than common commensal bacteria (Pei et al., 2024). Certainly, our study provides a substantial reservoir of high-quality novel phage sequences. This has important implications for exploring the compositional and functional diversity of pig gut prophages. This finding underscores the critical importance of mining prophage sequences from host genomes and providing novel perspectives for investigating the diverse interaction modalities between phages and their prokaryotic hosts through genomic context-driven discovery frameworks. In addition, understanding the relationship between prophages and their hosts provides valuable insights for regulating microbial networks in the pig gut. Such processes are critical for supporting gut health in the porcine.

Collectively, this study provides a systematic profiling of prophages and their distribution patterns and diversity within porcine gut prokaryotic hosts, highlighting their distinct phage-host interaction modalities. We provide an in-depth characterization of the functional roles of pig gut prophages, focusing on their interaction-mediated defense systems, prophage-mediated AMGs, ARGs, and VFs. Together, these findings highlight the multifaceted ways in which prophages shape host physiology, defense, and adaptation, offering critical insights into the ecological and evolutionary dynamics of the pig gut microbiome.

## 5 Conclusion

In conclusion, we conducted a comprehensive analysis of 10,742 prophage genomes identified from 7,524 bacterial and archaeal genomes of pig gut origin. This represents the first large-scale characterization of prophage diversity and host interactions in the pig gut microbiome. Our findings revealed substantial heterogeneity in prophage distribution across host species, with a subset exhibiting broad host range infectivity. Functional investigations highlighted the pivotal roles of prophages in enhancing host defense through CRISPR spacer acquisition and integration of immune-related genes. Additionally, prophages contributed to host adaptability by carrying auxiliary metabolic genes (notably those involved in vitamin B12 synthesis), antibiotic resistance genes, and virulence factors. Phylogenetic and functional analyses suggested that prophage-encoded integrases and tail proteins may influence host specificity. Finally, comparative analyses uncovered a rich reservoir of novel prophage sequences, significantly expanding the known diversity of the class Caudoviricetes, particularly within Crassvirales. Altogether, our study provides valuable insights into the ecological and functional roles of prophages in the pig gut microbiome and lays a foundational resource for future investigations into prophage-host dynamics in mammalian systems.

## Data Availability

The original contributions presented in the study are publicly available. This data can be found here: https://zenodo.org/records/15779497.

## Footnotes

^1^ http://vogdb.org

^2^ https://itol.embl.de/

^3^ http://www.mgc.ac.cn/VFs/

## Supplementary material

SUPPLEMENTARY FIGURE S1 The proportion of MH prophages, the distribution of prophages across the GC content and interactions, and the number of host genera for prophage genomes. **(a)** The proportion of MH (≥50% completeness) prophages at the genome level and species level (pie charts), and the number of MH prophages per prokaryotic genome (bar chart). **(b)** The distribution of identified prophages across the GC content of prokaryotic genomes. **(c)** The number distribution of interactions for prophage-prophage and host-prophage using spacer matching with different parameters. **(d)** The number of host genera targeted by each prophage, the number of spacers mapped to each prophage, and the proportion of prophages by CRISPR spacer matching.

SUPPLEMENTARY FIGURE S2 The representative examples for CRISPR-Cas systems and vitamin B12-related genes, and distribution of ARGs and VFs for prophage genomes. **(a)** Representative examples showing how prophages augment incomplete CRISPR-Cas systems in prokaryotic hosts. The light red and cyan horizontal solid lines represent prophage and host genomes regions, and the red vertical dotted lines represent the boundaries of prophage and host genomes predicted by VirSorter2. **(b)** The protein identity of vitamin B12-related genes for prophages and prokaryotic genomes. **(c)** The 3D structure of vitamin B12-related genes for prophages and prokaryotic genomes. **(d)** The vitamin B12 biosynthesis pathway and related genes. **(e)** The distribution of prophage-mediated ARGs across prophages with different infection host ranges. **(f)** The distribution of prophage-mediated VFGs across prophages with different host infection ranges.

## References

[ref1] Alcock B. P. Raphenya A. R. Lau T. T. Y. Tsang K. K. Bouchard M. Edalatmand A.. (2020). CARD 2020: antibiotic resistome surveillance with the comprehensive antibiotic resistance database. Nucleic Acids Res. 48, D517–D525. doi: 10.1093/nar/gkz935, PMID: 31665441 PMC7145624

[ref2] Al-Shayeb B. Sachdeva R. Chen L.-X. Ward F. Munk P. Devoto A.. (2020). Clades of huge phages from across Earth’s ecosystems. Nature 578, 425–431. doi: 10.1038/s41586-020-2007-4, PMID: 32051592 PMC7162821

[ref3] Altschu S. F. Gish W. Miller W. Myers E. W. Lipman D. J. (1990). Basic local alignment search tool. J. Mol. Biol. 215, 403–410. doi: 10.1016/S0022-2836(05)80360-2 2231712

[ref4] Bellas C. M. Schroeder D. C. Edwards A. Barker G. Anesio A. M. (2020). Flexible genes establish widespread bacteriophage pan-genomes in cryoconite hole ecosystems. Nat. Commun. 11:4403. doi: 10.1038/s41467-020-18236-8, PMID: 32879312 PMC7468147

[ref5] Benler S. Yutin N. Antipov D. Rayko M. Shmakov S. Gussow A. B.. (2021). Thousands of previously unknown phages discovered in whole-community human gut metagenomes. Microbiome 9:78. doi: 10.1186/s40168-021-01017-w, PMID: 33781338 PMC8008677

[ref6] Bernheim A. Sorek R. (2020). The pan-immune system of bacteria: antiviral defence as a community resource. Nat. Rev. Microbiol. 18, 113–119. doi: 10.1038/s41579-019-0278-2, PMID: 31695182

[ref7] Bondy-Denomy J. Davidson A. R. (2014). When a virus is not a parasite: the beneficial effects of prophages on bacterial fitness. J. Microbiol. 52, 235–242. doi: 10.1007/s12275-014-4083-3, PMID: 24585054

[ref8] Bondy-Denomy J. Qian J. Westra E. R. Buckling A. Guttman D. S. Davidson A. R.. (2016). Prophages mediate defense against phage infection through diverse mechanisms. ISME J. 10, 2854–2866. doi: 10.1038/ismej.2016.79, PMID: 27258950 PMC5148200

[ref9] Bosi E. Mascagni F. (2019). Less is more: genome reduction and the emergence of cooperation-implications into the coevolution of microbial communities. Int. J. Genomics 2019:2659175. doi: 10.1155/2019/2659175, PMID: 30911537 PMC6398007

[ref10] Brenes L. R. Laub M. T. (2025). *E. coli* prophages encode an arsenal of defense systems to protect against temperate phages. Cell Host Microbe 33, 1004–1018.e5. doi: 10.1016/j.chom.2025.04.021, PMID: 40409266

[ref11] Buchfink B. Xie C. Huson D. H. (2015). Fast and sensitive protein alignment using DIAMOND. Nat. Methods 12, 59–60. doi: 10.1038/nmeth.3176 25402007

[ref12] Camargo A. P. Roux S. Schulz F. Babinski M. Xu Y. Hu B.. (2023). Identification of mobile genetic elements with geNomad. Nat. Biotechnol. 42, 1303–1312. doi: 10.1038/s41587-023-01953-y, PMID: 37735266 PMC11324519

[ref13] Camarillo-Guerrero L. F. Almeida A. Rangel-Pineros G. Finn R. D. Lawley T. D. (2021). Massive expansion of human gut bacteriophage diversity. Cell 184, 1098–1109.e9. doi: 10.1016/j.cell.2021.01.029, PMID: 33606979 PMC7895897

[ref14] Capella-Gutierrez S. Silla-Martinez J. M. Gabaldon T. (2009). trimAl: a tool for automated alignment trimming in large-scale phylogenetic analyses. Bioinformatics 25, 1972–1973. doi: 10.1093/bioinformatics/btp348, PMID: 19505945 PMC2712344

[ref15] Chaumeil P. A. Mussig A. J. Hugenholtz P. Parks D. H. (2022). GTDB-Tk v2: memory friendly classification with the genome taxonomy database. Bioinformatics 38, 5315–5316. doi: 10.1093/bioinformatics/btac672, PMID: 36218463 PMC9710552

[ref16] Chen C. Fang S. Wei H. He M. Fu H. Xiong X.. (2021a). *Prevotella copri* increases fat accumulation in pigs fed with formula diets. Microbiome 9:175. doi: 10.1186/s40168-021-01110-0, PMID: 34419147 PMC8380364

[ref17] Chen Z. Yang H. Fu H. Wu L. Liu M. Jiang H.. (2022). Gut bacterial species in late trimester of pregnant sows influence the occurrence of stillborn piglet through pro-inflammation response. Front. Immunol. 13:1101130. doi: 10.3389/fimmu.2022.1101130, PMID: 36741405 PMC9890068

[ref18] Chen C. Zhou Y. Fu H. Xiong X. Fang S. Jiang H.. (2021b). Expanded catalog of microbial genes and metagenome-assembled genomes from the pig gut microbiome. Nat. Commun. 12:1106. doi: 10.1038/s41467-021-21295-0, PMID: 33597514 PMC7889623

[ref19] Chevallereau A. Pons B. J. van Houte S. Westra E. R. (2022). Interactions between bacterial and phage communities in natural environments. Nat. Rev. Microbiol. 20, 49–62. doi: 10.1038/s41579-021-00602-y, PMID: 34373631

[ref20] Chopin M.-C. Chopin A. Bidnenko E. (2005). Phage abortive infection in lactococci: variations on a theme. Curr. Opin. Microbiol. 8, 473–479. doi: 10.1016/j.mib.2005.06.006 15979388

[ref21] Clokie M. R. Millard A. D. Letarov A. V. Heaphy S. (2011). Phages in nature. Bacteriophage 1, 31–45. doi: 10.4161/bact.1.1.14942, PMID: 21687533 PMC3109452

[ref22] Couvin D. Bernheim A. Toffano-Nioche C. Touchon M. Michalik J. Neron B.. (2018). CRISPRCasFinder, an update of CRISRFinder, includes a portable version, enhanced performance and integrates search for Cas proteins. Nucleic Acids Res. 46, W246–W251. doi: 10.1093/nar/gky425, PMID: 29790974 PMC6030898

[ref23] Dance A. (2021). The incredible diversity of viruses. Nature 595, 22–25. doi: 10.1038/d41586-021-01749-7 34194016

[ref24] Dedrick R. M. Jacobs-Sera D. Bustamante C. A. Garlena R. A. Mavrich T. N. Pope W. H.. (2017). Prophage-mediated defence against viral attack and viral counter-defence. Nat. Microbiol. 2:16251. doi: 10.1038/nmicrobiol.2016.251, PMID: 28067906 PMC5508108

[ref25] Devoto A. E. Santini J. M. Olm M. R. Anantharaman K. Munk P. Tung J.. (2019). Megaphages infect Prevotella and variants are widespread in gut microbiomes. Nat. Microbiol. 4, 693–700. doi: 10.1038/s41564-018-0338-9, PMID: 30692672 PMC6784885

[ref26] Dominguez-Huerta G. Zayed A. A. Wainaina J. M. Guo J. Tian F. Pratama A. A.. (2022). Diversity and ecological footprint of Global Ocean RNA viruses. Science 376, 1202–1208. doi: 10.1126/science.abn6358, PMID: 35679415

[ref27] Feiner R. Argov T. Rabinovich L. Sigal N. Borovok I. Herskovits A. A. (2015). A new perspective on lysogeny: prophages as active regulatory switches of bacteria. Nat. Rev. Microbiol. 13, 641–650. doi: 10.1038/nrmicro3527, PMID: 26373372

[ref28] Fillol-Salom A. Bacigalupe R. Humphrey S. Chiang Y. N. Chen J. Penades J. R. (2021). Lateral transduction is inherent to the life cycle of the archetypical Salmonella phage P22. Nat. Commun. 12:6510. doi: 10.1038/s41467-021-26520-4, PMID: 34751192 PMC8575938

[ref29] Fu H. He M. Wu J. Zhou Y. Ke S. Chen Z.. (2021). Deep investigating the changes of gut microbiome and its correlation with the shifts of host serum metabolome around parturition in sows. Front. Microbiol. 12:729039. doi: 10.3389/fmicb.2021.729039, PMID: 34603257 PMC8484970

[ref30] Gregory A. C. Zablocki O. Zayed A. A. Howell A. Bolduc B. Sullivan M. B. (2020). The gut virome database reveals age-dependent patterns of virome diversity in the human gut. Cell Host Microbe 28, 724–740.e8. doi: 10.1016/j.chom.2020.08.003, PMID: 32841606 PMC7443397

[ref31] Guerin E. Shkoporov A. Stockdale S. R. Clooney A. G. Ryan F. J. Sutton T. D. S.. (2018). Biology and taxonomy of crAss-like bacteriophages, the most abundant virus in the human gut. Cell Host Microbe 24, 653–664.e6. doi: 10.1016/j.chom.2018.10.002, PMID: 30449316

[ref32] Guo J. Bolduc B. Zayed A. A. Varsani A. Dominguez-Huerta G. Delmont T. O.. (2021). VirSorter2: a multi-classifier, expert-guided approach to detect diverse DNA and RNA viruses. Microbiome 9:37. doi: 10.1186/s40168-020-00990-y, PMID: 33522966 PMC7852108

[ref33] Haft D. H. Selengut J. D. White O. (2003). The TIGRFAMs database of protein families. Nucleic Acids Res. 31, 371–373. doi: 10.1093/nar/gkg128, PMID: 12520025 PMC165575

[ref34] Harrison E. Brockhurst M. A. (2017). Ecological and evolutionary benefits of temperate phage: what does or doesn’t kill you makes you stronger. BioEssays 39:1700112. doi: 10.1002/bies.201700112, PMID: 28983932

[ref35] Holman D. B. Gzyl K. E. Mou K. T. Allen H. K. (2021). Weaning age and its effect on the development of the swine gut microbiome and resistome. mSystems 6:e0068221. doi: 10.1128/mSystems.00682-21, PMID: 34812652 PMC8609972

[ref36] Howard-Varona C. Hargreaves K. R. Abedon S. T. Sullivan M. B. (2017). Lysogeny in nature: mechanisms, impact and ecology of temperate phages. ISME J. 11, 1511–1520. doi: 10.1038/ismej.2017.16, PMID: 28291233 PMC5520141

[ref37] Hu J. Chen J. Ma L. Hou Q. Zhang Y. Kong X.. (2024a). Characterizing core microbiota and regulatory functions of the pig gut microbiome. ISME J. 18:wrad037. doi: 10.1093/ismejo/wrad037, PMID: 38366194 PMC10873858

[ref38] Hu J. Chen J. Nie Y. Zhou C. Hou Q. Yan X. (2024b). Characterizing the gut phageome and phage-borne antimicrobial resistance genes in pigs. Microbiome 12:102. doi: 10.1186/s40168-024-01818-9, PMID: 38840247 PMC11151549

[ref39] Hu J. Ye H. Wang S. Wang J. Han D. (2021). Prophage activation in the intestine: insights into functions and possible applications. Front. Microbiol. 12:785634. doi: 10.3389/fmicb.2021.785634, PMID: 34966370 PMC8710666

[ref40] Hurwitz B. L. U’Ren J. M. (2016). Viral metabolic reprogramming in marine ecosystems. Curr. Opin. Microbiol. 31, 161–168. doi: 10.1016/j.mib.2016.04.002, PMID: 27088500

[ref41] Hyatt D. Chen G. L. Locascio P. F. Land M. L. Larimer F. W. Hauser L. J. (2010). Prodigal: prokaryotic gene recognition and translation initiation site identification. BMC Bioinformatics 11:119. doi: 10.1186/1471-2105-11-119, PMID: 20211023 PMC2848648

[ref42] Jansen D. Matthijnssens J. (2023). The emerging role of the gut virome in health and inflammatory bowel disease: challenges, covariates and a viral imbalance. Viruses 15:173. doi: 10.3390/v15010173, PMID: 36680214 PMC9861652

[ref43] Jansson J. K. Wu R. (2022). Soil viral diversity, ecology and climate change. Nat. Rev. Microbiol. 21, 296–311. doi: 10.1038/s41579-022-00811-z, PMID: 36352025

[ref44] Jeudy S. Rigou S. Alempic J. M. Claverie J. M. Abergel C. Legendre M. (2020). The DNA methylation landscape of giant viruses. Nat. Commun. 11:2657. doi: 10.1038/s41467-020-16414-2, PMID: 32461636 PMC7253447

[ref45] Johansen J. Atarashi K. Arai Y. Hirose N. Sorensen S. J. Vatanen T.. (2023). Centenarians have a diverse gut virome with the potential to modulate metabolism and promote healthy lifespan. Nat. Microbiol. 8, 1064–1078. doi: 10.1038/s41564-023-01370-6, PMID: 37188814

[ref46] Jurėnas D. Fraikin N. Goormaghtigh F. Van Melderen L. (2022). Biology and evolution of bacterial toxin–antitoxin systems. Nat. Rev. Microbiol. 20, 335–350. doi: 10.1038/s41579-021-00661-1 34975154

[ref47] Katoh K. Standley D. M. (2013). MAFFT multiple sequence alignment software version 7: improvements in performance and usability. Mol. Biol. Evol. 30, 772–780. doi: 10.1093/molbev/mst010, PMID: 23329690 PMC3603318

[ref48] Kauffman K. M. Chang W. K. Brown J. M. Hussain F. A. Yang J. Polz M. F.. (2022). Resolving the structure of phage-bacteria interactions in the context of natural diversity. Nat. Commun. 13:372. doi: 10.1038/s41467-021-27583-z, PMID: 35042853 PMC8766483

[ref49] Kieft K. Zhou Z. Anantharaman K. (2020). VIBRANT: automated recovery, annotation and curation of microbial viruses, and evaluation of viral community function from genomic sequences. Microbiome 8:90. doi: 10.1186/s40168-020-00867-0, PMID: 32522236 PMC7288430

[ref50] Kieft K. Zhou Z. Anderson R. E. Buchan A. Campbell B. J. Hallam S. J.. (2021). Ecology of inorganic sulfur auxiliary metabolism in widespread bacteriophages. Nat. Commun. 12:3503. doi: 10.1038/s41467-021-23698-5, PMID: 34108477 PMC8190135

[ref51] Letunic I. Bork P. (2016). Interactive tree of life (iTOL) v3: an online tool for the display and annotation of phylogenetic and other trees. Nucleic Acids Res. 44, W242–W245. doi: 10.1093/nar/gkw290, PMID: 27095192 PMC4987883

[ref52] Liao H. Liu C. Zhou S. Liu C. Eldridge D. J. Ai C.. (2024). Prophage-encoded antibiotic resistance genes are enriched in human-impacted environments. Nat. Commun. 15:8315. doi: 10.1038/s41467-024-52450-y, PMID: 39333115 PMC11437078

[ref53] Lou Y. C. Chen L. Borges A. L. West-Roberts J. Firek B. A. Morowitz M. J.. (2024). Infant gut DNA bacteriophage strain persistence during the first 3 years of life. Cell Host Microbe 32, 35–47.e6. doi: 10.1016/j.chom.2023.11.015, PMID: 38096814 PMC11156429

[ref54] Makarova K. S. Wolf Y. I. Iranzo J. Shmakov S. A. Alkhnbashi O. S. Brouns S. J. J.. (2020). Evolutionary classification of CRISPR-Cas systems: a burst of class 2 and derived variants. Nat. Rev. Microbiol. 18, 67–83. doi: 10.1038/s41579-019-0299-x, PMID: 31857715 PMC8905525

[ref55] Mann N. H. Cook A. Millard A. Bailey S. Clokie M. (2003). Marine ecosystems: bacterial photosynthesis genes in a virus. Nature 424:741. doi: 10.1038/424741a, PMID: 12917674

[ref56] Markine-Goriaynoff N. Gillet L. Van Etten J. L. Korres H. Verma N. Vanderplasschen A. (2004). Glycosyltransferases encoded by viruses. J. Gen. Virol. 85, 2741–2754. doi: 10.1099/vir.0.80320-0, PMID: 15448335

[ref57] McGrath S. Fitzgerald G. F. van Sinderen D. (2002). Identification and characterization of phage-resistance genes in temperate lactococcal bacteriophages. Mol. Microbiol. 43, 509–520. doi: 10.1046/j.1365-2958.2002.02763.x, PMID: 11985726

[ref58] Medvedeva S. Sun J. Yutin N. Koonin E. V. Nunoura T. Rinke C.. (2022). Three families of Asgard archaeal viruses identified in metagenome-assembled genomes. Nat. Microbiol. 7, 962–973. doi: 10.1038/s41564-022-01144-6, PMID: 35760839 PMC11165672

[ref59] Mi J. Jing X. Ma C. Yang Y. Li Y. Zhang Y.. (2024). Massive expansion of the pig gut virome based on global metagenomic mining. npj Biofilms Microbiomes 10:76. doi: 10.1038/s41522-024-00554-0, PMID: 39209853 PMC11362615

[ref60] Middelboe M. Traving S. J. Castillo D. Kalatzis P. G. Glud R. N. (2025). Prophage-encoded chitinase gene supports growth of its bacterial host isolated from deep-sea sediments. ISME J. 19:wraf004. doi: 10.1093/ismejo/wraf004, PMID: 39832281 PMC11788074

[ref61] Mistry J. Chuguransky S. Williams L. Qureshi M. Salazar G. A. Sonnhammer E. L. L.. (2021). Pfam: the protein families database in 2021. Nucleic Acids Res. 49, D412–D419. doi: 10.1093/nar/gkaa913, PMID: 33125078 PMC7779014

[ref62] Nayfach S. Paez-Espino D. Call L. Low S. J. Sberro H. Ivanova N. N.. (2021). Metagenomic compendium of 189,680 DNA viruses from the human gut microbiome. Nat. Microbiol. 6, 960–970. doi: 10.1038/s41564-021-00928-6, PMID: 34168315 PMC8241571

[ref63] Neri U. Wolf Y. I. Roux S. Camargo A. P. Lee B. Kazlauskas D.. (2022). Expansion of the global RNA virome reveals diverse clades of bacteriophages. Cell 185, 4023–4037.e18. doi: 10.1016/j.cell.2022.08.023, PMID: 36174579

[ref64] Nobrega F. L. Vlot M. de Jonge P. A. Dreesens L. L. Beaumont H. J. E. Lavigne R.. (2018). Targeting mechanisms of tailed bacteriophages. Nat. Rev. Microbiol. 16, 760–773. doi: 10.1038/s41579-018-0070-8, PMID: 30104690

[ref65] Paez-Espino D. Pavlopoulos G. A. Ivanova N. N. Kyrpides N. C. (2017). Nontargeted virus sequence discovery pipeline and virus clustering for metagenomic data. Nat. Protoc. 12, 1673–1682. doi: 10.1038/nprot.2017.063, PMID: 28749930

[ref66] Parks D. H. Imelfort M. Skennerton C. T. Hugenholtz P. Tyson G. W. (2015). CheckM: assessing the quality of microbial genomes recovered from isolates, single cells, and metagenomes. Genome Res. 25, 1043–1055. doi: 10.1101/gr.186072.114, PMID: 25977477 PMC4484387

[ref67] Patel P. H. Taylor V. L. Zhang C. Getz L. J. Fitzpatrick A. D. Davidson A. R.. (2024). Anti-phage defence through inhibition of virion assembly. Nat. Commun. 15:1644. doi: 10.1038/s41467-024-45892-x, PMID: 38388474 PMC10884400

[ref68] Pei Z. Liu Y. Chen Y. Pan T. Sun X. Wang H.. (2024). A universe of human gut-derived bacterial prophages: unveiling the hidden viral players in intestinal microecology. Gut Microbes 16:2309684. doi: 10.1080/19490976.2024.2309684, PMID: 39679618 PMC10841027

[ref69] Pettersen E. F. Goddard T. D. Huang C. C. Meng E. C. Couch G. S. Croll T. I.. (2021). UCSF ChimeraX: structure visualization for researchers, educators, and developers. Protein Sci. 30, 70–82. doi: 10.1002/pro.3943, PMID: 32881101 PMC7737788

[ref70] Piel D. Bruto M. Labreuche Y. Blanquart F. Goudenège D. Barcia-Cruz R.. (2022). Phage–host coevolution in natural populations. Nat. Microbiol. 7, 1075–1086. doi: 10.1038/s41564-022-01157-1, PMID: 35760840

[ref71] Potter S. C. Luciani A. Eddy S. R. Park Y. Lopez R. Finn R. D. (2018). HMMER web server: 2018 update. Nucleic Acids Res. 46, W200–W204. doi: 10.1093/nar/gky448, PMID: 29905871 PMC6030962

[ref72] Price M. N. Dehal P. S. Arkin A. P. (2010). FastTree 2—approximately maximum-likelihood trees for large alignments. PLoS One 5:e9490. doi: 10.1371/journal.pone.0009490, PMID: 20224823 PMC2835736

[ref73] Rao L. Cai L. Huang L. (2023). Single-cell dynamics of liver development in postnatal pigs. Sci. Bull. 68, 2583–2597. doi: 10.1016/j.scib.2023.09.021, PMID: 37783617

[ref74] Schulz F. Roux S. Paez-Espino D. Jungbluth S. Walsh D. A. Denef V. J.. (2020). Giant virus diversity and host interactions through global metagenomics. Nature 578, 432–436. doi: 10.1038/s41586-020-1957-x, PMID: 31968354 PMC7162819

[ref75] Shaffer M. Borton M. A. McGivern B. B. Zayed A. A. La Rosa S. L. Solden L. M.. (2020). DRAM for distilling microbial metabolism to automate the curation of microbiome function. Nucleic Acids Res. 48, 8883–8900. doi: 10.1093/nar/gkaa621, PMID: 32766782 PMC7498326

[ref76] Shkoporov A. N. Stockdale S. R. Lavelle A. Kondova I. Heuston C. Upadrasta A.. (2022). Viral biogeography of the mammalian gut and parenchymal organs. Nat. Microbiol. 7, 1301–1311. doi: 10.1038/s41564-022-01178-w, PMID: 35918425 PMC7614033

[ref77] Steinegger M. Soding J. (2017). MMseqs2 enables sensitive protein sequence searching for the analysis of massive data sets. Nat. Biotechnol. 35, 1026–1028. doi: 10.1038/nbt.3988, PMID: 29035372

[ref78] Sutcliffe S. G. Reyes A. Maurice C. F. (2023). Bacteriophages playing nice: lysogenic bacteriophage replication stable in the human gut microbiota. iScience 26:106007. doi: 10.1016/j.isci.2023.106007, PMID: 36798434 PMC9926308

[ref79] Tan D. Hansen M. F. de Carvalho L. N. Roder H. L. Burmolle M. Middelboe M.. (2020). High cell densities favor lysogeny: induction of an H20 prophage is repressed by quorum sensing and enhances biofilm formation in *Vibrio anguillarum*. ISME J. 14, 1731–1742. doi: 10.1038/s41396-020-0641-3, PMID: 32269377 PMC7305317

[ref80] Touchon M. Bernheim A. Rocha E. P. (2016). Genetic and life-history traits associated with the distribution of prophages in bacteria. ISME J. 10, 2744–2754. doi: 10.1038/ismej.2016.47, PMID: 27015004 PMC5113838

[ref81] Tunyasuvunakool K. Adler J. Wu Z. Green T. Zielinski M. Zidek A.. (2021). Highly accurate protein structure prediction for the human proteome. Nature 596, 590–596. doi: 10.1038/s41586-021-03828-1, PMID: 34293799 PMC8387240

[ref82] Uc-Mass A. Loeza E. J. de la Garza M. Guarneros G. Hernandez-Sanchez J. Kameyama L. (2004). An orthologue of the *cor* gene is involved in the exclusion of temperate lambdoid phages. Evidence that Cor inactivates FhuA receptor functions. Virology 329, 425–433. doi: 10.1016/j.virol.2004.09.005, PMID: 15518820

[ref83] Warwick-Dugdale J. Buchholz H. H. Allen M. J. Temperton B. (2019). Host-hijacking and planktonic piracy: how phages command the microbial high seas. Virol. J. 16:15. doi: 10.1186/s12985-019-1120-1, PMID: 30709355 PMC6359870

[ref84] Waterhouse R. M. Seppey M. Simao F. A. Manni M. Ioannidis P. Klioutchnikov G.. (2018). BUSCO applications from quality assessments to gene prediction and Phylogenomics. Mol. Biol. Evol. 35, 543–548. doi: 10.1093/molbev/msx319, PMID: 29220515 PMC5850278

[ref85] Wendling C. C. (2023). Prophage mediated control of higher order interactions—insights from multi-level approaches. Curr. Opin. Syst. Biol. 35:100469. doi: 10.1016/j.coisb.2023.100469

[ref86] Wendling C. C. Refardt D. Hall A. R. (2021). Fitness benefits to bacteria of carrying prophages and prophage-encoded antibiotic-resistance genes peak in different environments. Evolution 75, 515–528. doi: 10.1111/evo.14153, PMID: 33347602 PMC7986917

[ref87] Wienhausen G. Moraru C. Bruns S. Tran D. Q. Sultana S. Wilkes H.. (2024). Ligand cross-feeding resolves bacterial vitamin B_12_ auxotrophies. Nature 629, 886–892. doi: 10.1038/s41586-024-07396-y, PMID: 38720071

[ref88] Wu Z. Liu S. Ni J. (2024). Metagenomic characterization of viruses and mobile genetic elements associated with the DPANN archaeal superphylum. Nat. Microbiol. 9, 3362–3375. doi: 10.1038/s41564-024-01839-y, PMID: 39448846

[ref89] Yan M. Pratama A. A. Somasundaram S. Li Z. Jiang Y. Sullivan M. B.. (2023). Interrogating the viral dark matter of the rumen ecosystem with a global virome database. Nat. Commun. 14:5254. doi: 10.1038/s41467-023-41075-2, PMID: 37644066 PMC10465536

[ref90] Yang H. Wu J. Huang X. Zhou Y. Zhang Y. Liu M.. (2022). ABO genotype alters the gut microbiota by regulating GalNAc levels in pigs. Nature 606, 358–367. doi: 10.1038/s41586-022-04769-z, PMID: 35477154 PMC9157047

[ref91] Yang B. Yang J. Chen R. Chai J. Wei X. Zhao J.. (2024). Metagenome-assembled genomes of pig fecal samples in nine European countries: insights into antibiotic resistance genes and viruses. Microorganisms 12:2409. doi: 10.3390/microorganisms12122409, PMID: 39770612 PMC11676251

[ref92] Yehl K. Lemire S. Yang A. C. Ando H. Mimee M. Torres M. T.. (2019). Engineering phage host-range and suppressing bacterial resistance through phage tail fiber mutagenesis. Cell 179, 459–469.e9. doi: 10.1016/j.cell.2019.09.015 31585083 PMC6924272

[ref93] Yi Y. Liu S. Hao Y. Sun Q. Lei X. Wang Y.. (2023). A systematic analysis of marine lysogens and proviruses. Nat. Commun. 14:6013. doi: 10.1038/s41467-023-41699-4, PMID: 37758717 PMC10533544

[ref94] Yin Z. Zhang S. Wei Y. Wang M. Ma S. Yang S.. (2016). Horizontal gene transfer clarifies taxonomic confusion and promotes the genetic diversity and pathogenicity of *Plesiomonas shigelloides*. ISME J. 10, 2787–2800. doi: 10.1128/mSystems.00448-20 32934114 PMC7498682

[ref95] Yu M. Chu Y. Wang Y. Mo L. Tan X. Guo S.. (2025). Metagenomic analysis reveals gut phage diversity across three mammalian models. Microbiome 13:146. doi: 10.1186/s40168-025-02144-4, PMID: 40542420 PMC12180220

[ref96] Yutin N. Benler S. Shmakov S. A. Wolf Y. I. Tolstoy I. Rayko M.. (2021). Analysis of metagenome-assembled viral genomes from the human gut reveals diverse putative CrAss-like phages with unique genomic features. Nat. Commun. 12:1044. doi: 10.1038/s41467-021-21350-w, PMID: 33594055 PMC7886860

[ref97] Zeng Z. Liu X. Yao J. Guo Y. Li B. Li Y.. (2016). Cold adaptation regulated by cryptic prophage excision in *Shewanella oneidensis*. ISME J. 10, 2787–2800. doi: 10.1038/ismej.2016.85, PMID: 27482926 PMC5148205

