# Supplementary figures and images for: Comprehensive discovery and functional characterization of diverse prophages in the pig gut microbiome
Source: Front Microbiol. 2025 Sep 9;16:1662087. doi: 10.3389/fmicb.2025.1662087 (PMC12454387; doi:10.3389/fmicb.2025.1662087)

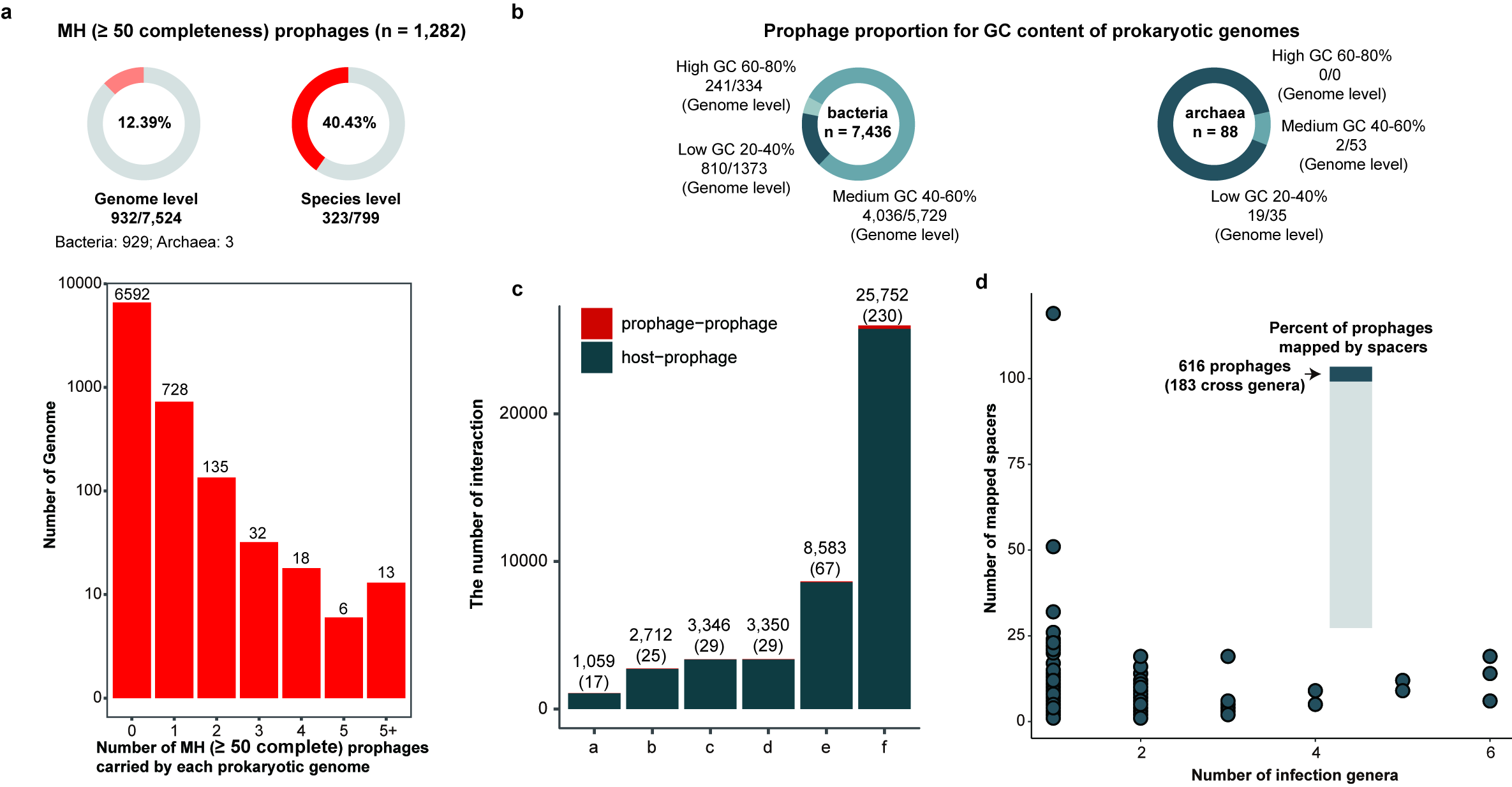

Supplement: SUPPLEMENTARY FIGURE S1 — The proportion of MH prophages, the distribution of prophages across the GC content and interactions, and the number of host genera for prophage genomes. (a) The proportion of MH (≥50% completeness) prophages at the genome level and species level (pie charts), and the number of MH prophages per prokaryotic genome (bar chart). (b) The distribution of identified prophages across the GC content of prokaryotic genomes. (c) The number distribution of interactions for prophage-prophage and host-prophage using spacer matching with different parameters. (d) The number of host genera targeted by each prophage, the number of spacers mapped to each prophage, and the proportion of prophages by CRISPR spacer matching. [file Image_1.TIF]

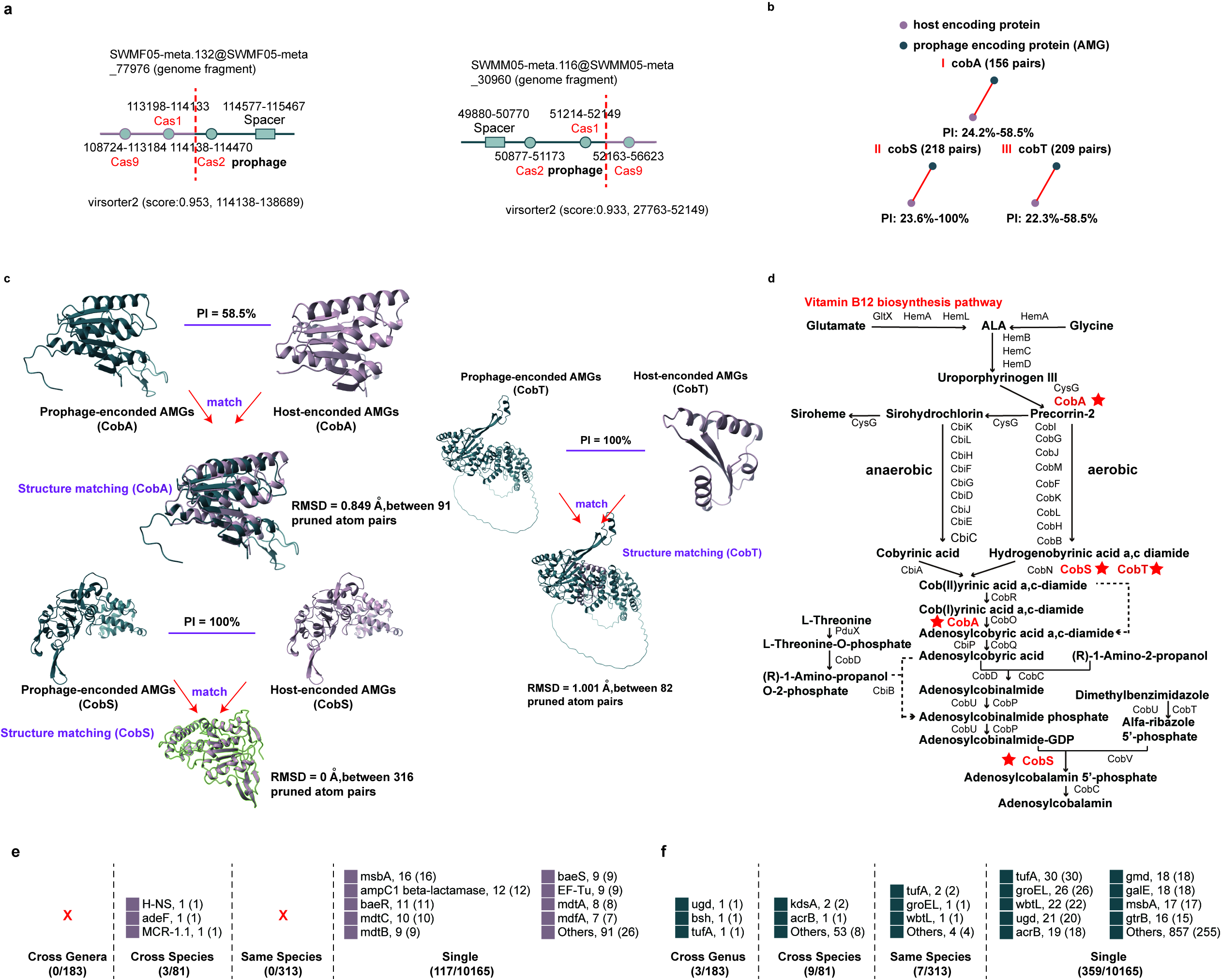

Supplement: SUPPLEMENTARY FIGURE S2 — The representative examples for CRISPR-Cas systems and vitamin B12-related genes, and distribution of ARGs and VFs for prophage genomes. (a) Representative examples showing how prophages augment incomplete CRISPR-Cas systems in prokaryotic hosts. The light red and cyan horizontal solid lines represent prophage and host genomes regions, and the red vertical dotted lines represent the boundaries of prophage and host genomes predicted by VirSorter2. (b) The protein identity of vitamin B12-related genes for prophages and prokaryotic genomes. (c) The 3D structure of vitamin B12-related genes for prophages and prokaryotic genomes. (d) The vitamin B12 biosynthesis pathway and related genes. (e) The distribution of prophage-mediated ARGs across prophages with different infection host ranges. (f) The distribution of prophage-mediated VFGs across prophages with different host infection ranges. [file Image_2.TIF]
